# LINC-EPS Protects Against Neurodegeneration by Driving a PGC-1α-Mediated Anti-Ferroptosis Program in Parkinson's Disease

**DOI:** 10.7150/ijbs.128204

**Published:** 2026-03-17

**Authors:** Ziqi Liu, Ruoxun Wang, Xinrui Lan, Min Shen, Mingfeng Jiang, Rongqing Li, Jie Zhao, Jing Li, Sainan Wang, Qicheng Wang, Xinyi Xu, Wei Li, Weijuan Gong, Li Qian

**Affiliations:** 1 Key Laboratory of the Jiangsu Higher Education Institutions for Nucleic Acid & Cell Fate Regulation (Yangzhou University), Faculty of Medicine, Yangzhou University, Yangzhou, Jiangsu, 225001, PR China.; 2 Department of Center Laboratory, Kunshan Hospital of Chinese Medicine, Affiliated Hospital of Yangzhou University, Kunshan, Jiangsu, 215300, PR China.

**Keywords:** Parkinson's disease, LINC-EPS, PGC-1α, ferroptosis, mitochondria, neurodegeneration

## Abstract

Parkinson's disease (PD) is a progressive neurodegenerative disorder characterized by dopaminergic (DA) neuron loss and currently lacks disease-modifying treatments. We found that the long intergenic non-coding RNA LINC-EPS was markedly reduced in peripheral blood of PD patients, correlating with greater clinical severity. Similar downregulation was observed in 1-methyl-4-phenyl-1,2,3,6-tetrahydropyridine (MPTP) PD mice and 1-methyl-4-phenylpyridinium-treated DA neurons. Knockout of LINC-EPS, either systemically or specifically in DA neurons, aggravated motor deficits and DA neurodegeneration, whereas AAV-mediated overexpression rescued these phenotypes. LINC-EPS protected DA neurons by suppressing ferroptosis, acting as a scaffold that binds both PGC-1α protein and a T-box element in its promoter, thereby recruiting PGC-1α to enhance its own transcription through a positive feedback loop. This activation improved mitochondrial function, lowered reactive oxygen species, inhibited lipid peroxidation, and conferred ferroptosis resistance. Pharmacological activation of PGC-1α with ZLN005 rescued neurodegeneration in LINC-EPS-deficient PD mice. Our study identifies a novel LINC-EPS/PGC-1α axis that mitigates ferroptotic DA neuron loss and supports PGC-1α activation as a promising therapeutic strategy for PD progression.

## Introduction

Parkinson's disease (PD) is a progressive neurodegenerative disorder characterized by the selective loss of dopaminergic (DA) neurons in the substantia nigra (SN) pars compacta (SNpc) and profound depletion of striatal dopamine (Str). Disruption of the nigrostriatal pathway culminates in cardinal motor symptoms, including bradykinesia, rigidity, resting tremors, and postural instability 1, 2. Current therapeutic strategies, although effective in managing symptoms, are largely symptomatic and do not halt or reverse the underlying neurodegenerative processes. This therapeutic impasse underscores the critical gap in our understanding of the molecular mechanisms driving neuronal vulnerability in PD 3.

DA neurons in SNpc are particularly vulnerable to PD, which is intrinsically linked to their high metabolic demand and reliance on mitochondrial integrity. Mitochondrial dysfunction, a well-established hallmark of PD, results in a bioenergetic crisis leading to ATP depletion, impaired calcium homeostasis, and accumulation of damaged organelles, which collectively compromise neuronal survival 4-6. Furthermore, compromised mitochondrial respiratory chain function, particularly in complex I, results in the excessive production of mitochondrial reactive oxygen species (mitoROS) 7-9. This enhanced oxidative stress not only damages cellular macromolecules but also creates conditions that render neurons particularly susceptible to iron-dependent lipid peroxidation, thereby linking mitochondrial dysfunction to ferroptotic cell death 10, 11.

Ferroptosis, an iron-dependent form of regulated cell death driven by lipid peroxidation, emerges as a critical mechanism in PD pathogenesis. SNpc is characterized by a high iron content, and DA neurons are enriched with polyunsaturated fatty acids, rendering them extremely sensitive to ferroptotic insults 12-14. Critically, mitochondrial dysfunction directly drives ferroptosis: overproduction of mitoROS, in combination with iron-catalyzed Fenton chemistry, triggers catastrophic lipid peroxidation 15-17. This is further exacerbated by the intrinsic properties of dopamine metabolism, which inactivates key antioxidant defenses, such as glutathione peroxidase 4 (GPX4), dismantling the primary cellular defense against ferroptotic death 18, 19. Thus, the mitochondrial dysfunction-ferroptosis axis represents a mechanistically integrated pathway central to DA neuron degeneration in PD.

Long non-coding RNAs (lncRNAs) serve as critical regulators of diverse cellular processes, including neuronal function and survival, and often act as molecular scaffolds or transcriptional modulators 20-22. Specifically, long intergenic non-coding RNA EPS (*LINC-EPS*) is primarily known for its role in erythropoiesis and as a systemic anti-inflammatory regulator of immune cells. It acts as a protective factor for neuronal cells against cerebral ischemia. *LINC-EPS* exerts neuroprotective effects on neurons exposed to oxygen-glucose deprivation/reoxygenation by modulating the Sir1-mediated regulation of autophagy, thereby influencing the neuroinflammatory responses and apoptotic pathways 23. However, whether LINC-EPS is involved in the pathogenesis of chronic neurodegenerative disorders, such as PD, and the molecular mechanisms by which it exerts neuroprotective effects remain unknown.

Here, we demonstrate that LINC-EPS, preferentially expressed in the SN, is significantly reduced in PD patients and correlates with disease severity. Using both systemic and cell-type-specific genetic approaches in MPTP-induced PD models, we show that LINC-EPS deficiency exacerbates dopaminergic neurodegeneration, while its overexpression confers neuroprotection. Mechanistically, LINC-EPS maintains peroxisome proliferator-activated receptor-γ coactivator-1α (PGC-1α) expression to preserve mitochondrial homeostasis and suppress ferroptosis-mediated neuronal death 24, 25. Pharmacological PGC-1α activation rescues the neurodegenerative phenotypes in LINC-EPS-deficient mice, establishing a LINC-EPS/PGC-1α axis as a potential therapeutic target for PD 25, 26.

## Materials and Methods

### Reagents and antibodies

Comprehensive lists of key reagents, kits, and antibodies used in this study are provided in the Supplementary Materials (Table S1-S2).

### Human clinical specimens and ethical statement

Peripheral blood samples were obtained from 60 participants, including 30 patients with PD and 30 healthy controls. Clinical demographics and disease characteristics, including age, gender, disease duration, and Unified Parkinson's Disease Rating Scale III (UPDRS-III) scores, are detailed in the Supplementary Materials (Table S3). This study was approved by The Ethics Committee of Yangzhou University School of Medicine (Approval No. YXYLL-2025-106) and conducted in adherence to the Declaration of Helsinki. Written informed consent was obtained from all participants, and samples were anonymized to ensure privacy.

### Animals and ethical statement

All animal experiments were conducted in compliance with protocols approved by the Institutional Animal Care and Use Committee (IACUC) of Yangzhou University Medical College. Male C57BL/6J wild-type mice (8-10 weeks old, 25 ± 2 g) were sourced from the Comparative Medicine Centre of Yangzhou University. LINC-EPS knockout (*LINC-EPS^⁻/⁻^*) mice on a C57BL/6J background were generously provided by Dr. Feng Ma (Institute of Systems Medicine, Chinese Academy of Medical Sciences), while LINC-EPS floxed (*LINC-EPS^fl/fl^*) and DAT-Cre mice were custom-generated by Cyagen Biosciences (Suzhou, China). Animals were housed in a specific-pathogen-free (SPF) environment and maintained on a 12-hour light/dark cycle at a controlled temperature (20 ± 2°C), with *ad libitum* access to standard chow and water. Genotyping was performed by PCR analysis of tail DNA; the specific primers and PCR protocols are detailed in the Supplementary Materials (Table S4).

### Cell lines and culture

The cell lines utilized in this study included human neuroblastoma SH-SY5Y, murine microglial BV2, murine astrocyte C8-D1A, and human embryonic kidney (HEK) 293T. The identity of the SH-SY5Y line was authenticated via short tandem repeat (STR) profiling, and all cultures were routinely confirmed to be negative for mycoplasma contamination using PCR-based assays. Cells were maintained in high-glucose Dulbecco's Modified Eagle's Medium (DMEM; Servicebio) supplemented with 10% fetal bovine serum (FBS; Gibco) and 1% penicillin-streptomycin (Beyotime). For lentiviral production, HEK293T cells were cultured in Opti-MEM Reduced Serum Medium (Gibco). All cultures were maintained in a humidified incubator at 37°C with a 5% CO₂ atmosphere.

### Primary neuron and glial cultures

Primary midbrain neurons were prepared from postnatal day 0-1 (P0-P1) C57BL/6J mouse pups. Briefly, midbrain tissues were dissected, dissociated with papain (2 mg/mL; Worthington) and DNase I (0.1 mg/mL; Sigma-Aldrich), and plated on poly-D-lysine-coated plates. Neurons were cultured in Neurobasal-A medium supplemented with B27 (Gibco) and GlutaMAX (Gibco). Cytosine arabinoside (Ara-C; 5 µM; Sigma-Aldrich) was added at 24 h post-plating to inhibit glial proliferation 27.

For mixed glial cultures, cerebral cortices from P0-P1 pups were dissociated with 0.125% trypsin. Cells were plated in DMEM/F12 with 10% FBS. After 14-20 days, microglia were isolated by shaking the flasks at 200 rpm for 4 h. The adherent cells remaining in the flask were cultured as primary astrocytes 28.

### Parkinson's disease models

*In vivo* PD models were established by administering MPTP (30 mg/kg, i.p.; Sigma-Aldrich) daily for 5 consecutive days. Control mice received equivalent volumes of sterile saline. For genetic models, WT and *LINC-EPS*^⁻/⁻^ mice, as well as *LINC-EPS*^flox/flox^ and DAT-Cre; *LINC-EPS*^flox/flox^ (*LINC-EPS*^ΔDat^) mice, were randomly assigned to saline or MPTP treatment groups (n = 10-12 mice per group).

*In vitro* PD models were established by treating primary midbrain neurons or SH-SY5Y cells with 500 µM MPP⁺ (Sigma-Aldrich), the active toxic metabolite of MPTP, for 24 h 29, 30.

### Behavioral analyses

Mice were habituated to the testing room for at least 30 min before each test.

* Pole Test:* Mice were placed head-up on top of a vertical wooden pole (50 cm height, 1 cm diameter). The time to turn and descend to the floor was recorded.

* Open Field Test:* Spontaneous activity was recorded for 5 min in an open field arena (40 cm × 40 cm × 40 cm). Total distance, average speed, and time immobile were analyzed using an automated tracking system (ANY-maze).

* Rotarod Test:* Motor coordination was assessed on an accelerating rotarod (Ugo Basile). The rod accelerated from 4 to 40 rpm over 300 s. The latency to fall was recorded across three trials per day for three consecutive days 31.

### Computational modeling of molecular interactions

#### Protein-RNA docking

To predict the binding interface between the PGC-1α protein and LINC-EPS RNA, their respective three-dimensional structures were first generated using AlphaFold3. Molecular docking simulations were subsequently performed using the HDOCK server. PGC-1α was designated as the receptor and LINC-EPS RNA as the ligand, with docking parameters set to a grid spacing of 1.200 and an angular step of 15.000. The conformation with the optimal docking score was selected for visualization in PyMOL, where key hydrogen bond interactions between PGC-1α amino acid residues and LINC-EPS nucleotides were identified and analyzed.

#### RNA-DNA triplex modeling

To investigate the potential formation of an RNA-DNA triplex, a computational model was constructed to simulate the interaction between LINC-EPS RNA and the double-stranded DNA sequence of the TBE. The predicted triplex structure was visualized in PyMOL to map the specific atomic interactions, including the Hoogsteen base pairing, that stabilize the complex 31-33.

#### RNA extraction and sequencing library preparation

Total RNA was extracted from dissected mouse SN tissues using TRIzol® Reagent (Magen). The concentration and purity of the extracted RNA were measured using a NanoDrop ND-2000 spectrophotometer, while its integrity was validated on an Agilent Bioanalyzer 4150 system. Only high-quality RNA samples with an RNA Integrity Number (RIN) of 7.0 or greater were processed for library construction. Paired-end sequencing libraries were prepared using the VAHTS Universal V6 RNA-seq Library Prep Kit (Vazyme). Briefly, polyadenylated (poly(A)) mRNA was enriched from total RNA using oligo(dT)-conjugated magnetic beads, subjected to thermal fragmentation, and then reverse-transcribed into first-strand cDNA using random hexamer primers. Following second-strand synthesis and adapter ligation, the resulting cDNA fragments were enriched by PCR amplification. The final libraries were purified and their quality confirmed on an Agilent Bioanalyzer 4150. Qualified libraries were then sequenced on the Illumina NovaSeq 6000 platform, generating 150-bp paired-end reads.

### Statistical analysis

Data are presented as mean ± SEM. Statistical analyses were performed using GraphPad Prism 10.0 (GraphPad Software). Comparisons between two groups were made using a two-tailed Student's t-test. For comparisons among multiple groups, one-way ANOVA and two-way ANOVA followed by Tukey's multiple comparison test was used. Correlations between two continuous variables were assessed using Pearson's correlation coefficient analysis. A P-value < 0.05 was considered statistically significant (*P < 0.05, **P < 0.01, ***P < 0.001).

## Results

### LINC-EPS expression is downregulated in PD and its deficiency exacerbates nigrostriatal neuropathology

**Figure 1:**
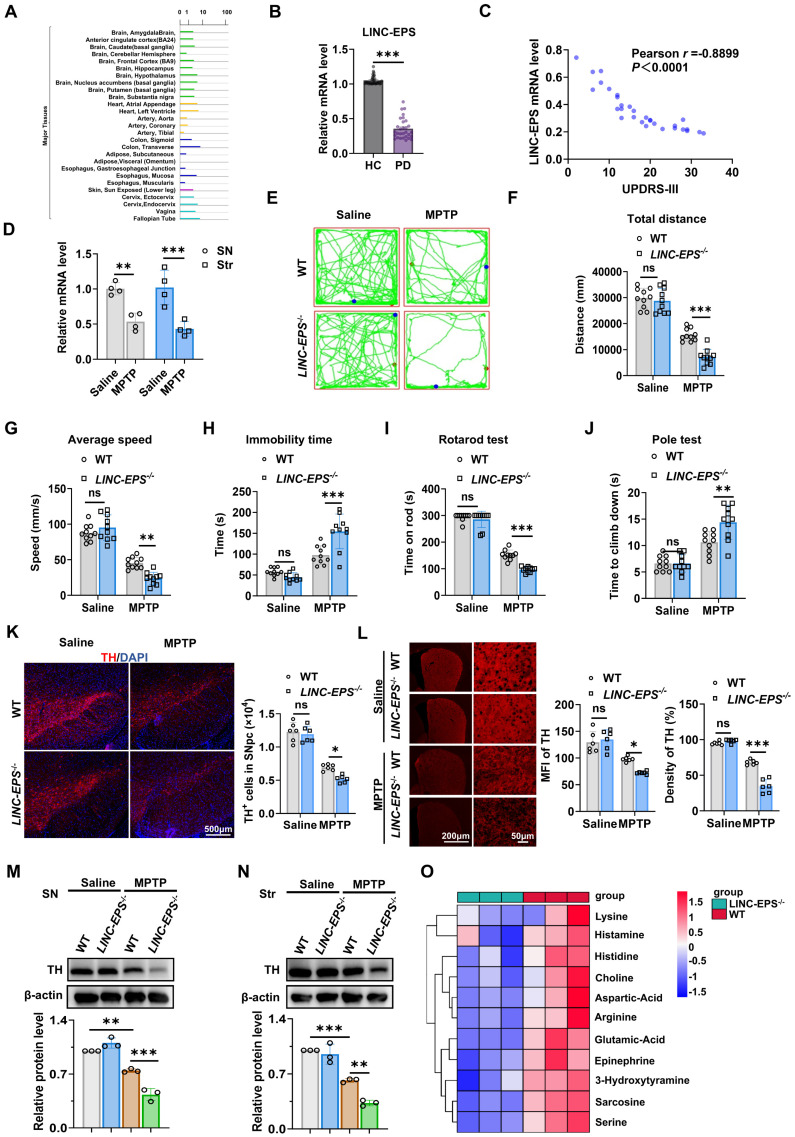
** LINC-EPS Deficiency Exacerbates MPTP-Induced Neurodegeneration. (A)** Bioinformatic analysis of LINC-EPS mRNA expression across a panel of human tissues using the GTEx database. The SN is indicated to show its notable expression level. **(B)** RT-qPCR analysis of *LINC-EPS* mRNA levels in peripheral blood samples from healthy controls (HC) (n = 30) and PD patients (n = 30). **(C)** Pearson correlation analysis of peripheral *LINC-EPS* mRNA levels and UPDRS-III motor scores in PD patients (n = 30). **(D)** RT-qPCR analysis of *LINC-EPS* mRNA levels in the Substantia Nigra (SN) and striatum (Str) of C57/BL6 mice treated with saline or MPTP (n = 4). **(E-J)** Motor function assessments: open-field test parameters **(E-H)**, rotarod performance **(I)**, and pole test **(J)** (n=10). **(K)** Representative immunofluorescence images of TH (red) in the SNpc and quantification of TH-positive (TH⁺) neurons. DAPI (blue) was used for nuclear counterstaining. Scale bar, 500 µm (n = 6). **(L)** Representative immunofluorescence images showing TH-positive fibers in the striatum from saline- or MPTP-treated wild-type (WT) and *LINC-EPS^⁻/⁻^* mice, together with quantification of mean fluorescence intensity (MFI) and relative density. Scale bars, 200 µm (overview) and 50 µm (magnified view) (n = 6). **(M-N)** Representative immunoblots and quantification of TH protein levels in the SN **(M)** and striatum** (N)** β-actin served as a loading control. Data are presented as fold change, where the ratio of TH to β-actin in the control group was designated as 1.0 (n = 3). **(O)** Metabolomic alterations in LINC-EPS^⁻/⁻^ striatum following MPTP treatment (n=3). Data are presented as mean ± SEM. Statistical significance was determined by two-tailed unpaired Student's t-test for **(B)**, Pearson correlation for **(C)**, two-way ANOVA with Tukey's post-hoc test for **(D, F-L)**, and one-way ANOVA with Tukey's post-hoc test for **(M, N)**. *P < 0.05, **P < 0.01, ***P < 0.001. ns, not significant.

Analysis of the Genotype-Tissue Expression (GTEx) dataset revealed LINC-EPS expression in human SN (Fig. 1A) 34. To assess a clinically accessible biospecimen, we quantified LINC-EPS expression in peripheral blood from an independent cohort by RT-qPCR, revealing significantly lower levels in PD patients compared with healthy controls (Fig. 1B). To evaluate the clinical significance of this reduction, correlations between peripheral *LINC-EPS* mRNA levels and motor symptom severity were examined. A significant inverse relationship was observed between LINC-EPS expression levels and the Unified PD Rating Scale part III (UPDRS-III) motor scores (Fig. 1C), indicating that low peripheral LINC-EPS expression was associated with high motor impairment. Consistent with the findings in human peripheral blood, mRNA levels of LINC-EPS were significantly reduced in the SN and striatum of MPTP-induced PD model mice (Fig. 1D).

We subjected global *LINC-EPS* knockout (*LINC-EPS*^⁻/⁻^) mice and their wild-type (WT) littermates to the MPTP regimen to determine the role of LINC-EPS in PD-like pathology 35. MPTP-treated *LINC-EPS*^⁻/⁻^ mice exhibited more severe motor deficits than their WT counterparts. In the open-field test, these mice traveled a shorter total distance, showed a lower average speed, and spent significantly more time immobile (Fig. 1E-H). In the accelerating rotarod test, these mice exhibited a shorter latency to fall (Fig. 1I). Furthermore, these mice showed prolonged descent time in the pole test, consistent with exacerbated bradykinesia (Fig. 1J).

Consistent with the exacerbated behavioral deficits, *LINC-EPS* deficiency aggravated MPTP-induced nigrostriatal neurodegeneration. Immunofluorescence analysis for tyrosine hydroxylase (TH) in SNpc revealed the significantly greater loss of TH-positive neurons in MPTP-treated *LINC-EPS^⁻/⁻^* mice than in MPTP-treated WT controls (Fig. 1K). Quantitative analysis confirmed the significantly greater reduction in mean fluorescence intensity and TH-immunoreactive fiber density in the striatum of *LINC-EPS^⁻/⁻^* mice than in the striatum of their WT littermates following MPTP administration (Fig. 1L). These neuropathological findings were confirmed via immunoblotting, which indicated significantly lower TH protein levels in the SN and striatum of MPTP-treated *LINC-EPS^⁻/⁻^* mice compared to those in the SN and striatum of WT controls (Fig. 1M and N).

We performed targeted metabolomic analysis of striatal tissue to quantify the neurochemical state of the nigrostriatal system. This analysis revealed a more pronounced depletion of key neurotransmitters in *LINC-EPS^⁻/⁻^* mice than in their WT counterparts following MPTP administration. Specifically, striatal levels of 3-hydroxytyramine (dopamine) and epinephrine were markedly reduced in *LINC-EPS*-deficient animals. Furthermore, a global reduction was observed across multiple neuroactive metabolites, including excitatory transmitters glutamic acid and aspartic acid, and the cholinergic metabolite choline (Fig. 1O). These data suggest that *LINC-EPS* deficiency causes a profound collapse of striatal neurochemical homeostasis in the Parkinsonian state. Furthermore, expression levels of key neurotrophic factors, such as brain-derived neurotrophic factor (Bdnf) and neurotrophin-3 (Nt3), were significantly lower in the midbrain of MPTP-treated *LINC-EPS*^⁻/⁻^ mice (Fig. S1A). We further examined the accumulation of α-synuclein phosphorylated at serine 129 (pS129-α-Syn), a core pathological hallmark of PD 36. Both immunofluorescence and immunoblotting assays revealed the significantly greater accumulation of pS129-α-Syn in the nigrostriatal system of MPTP-treated *LINC-EPS^⁻/⁻^* mice compared to that in the nigrostriatal system of MPTP-treated WT controls (Fig. S1B-E).

Collectively, these data indicate that LINC-EPS deficiency results in severe motor deficits, DA neurodegeneration, and α-synuclein pathology in MPTP induced PD model mice.

### LINC-EPS deficiency in DA neurons exacerbates nigrostriatal neurodegeneration

**Figure 2:**
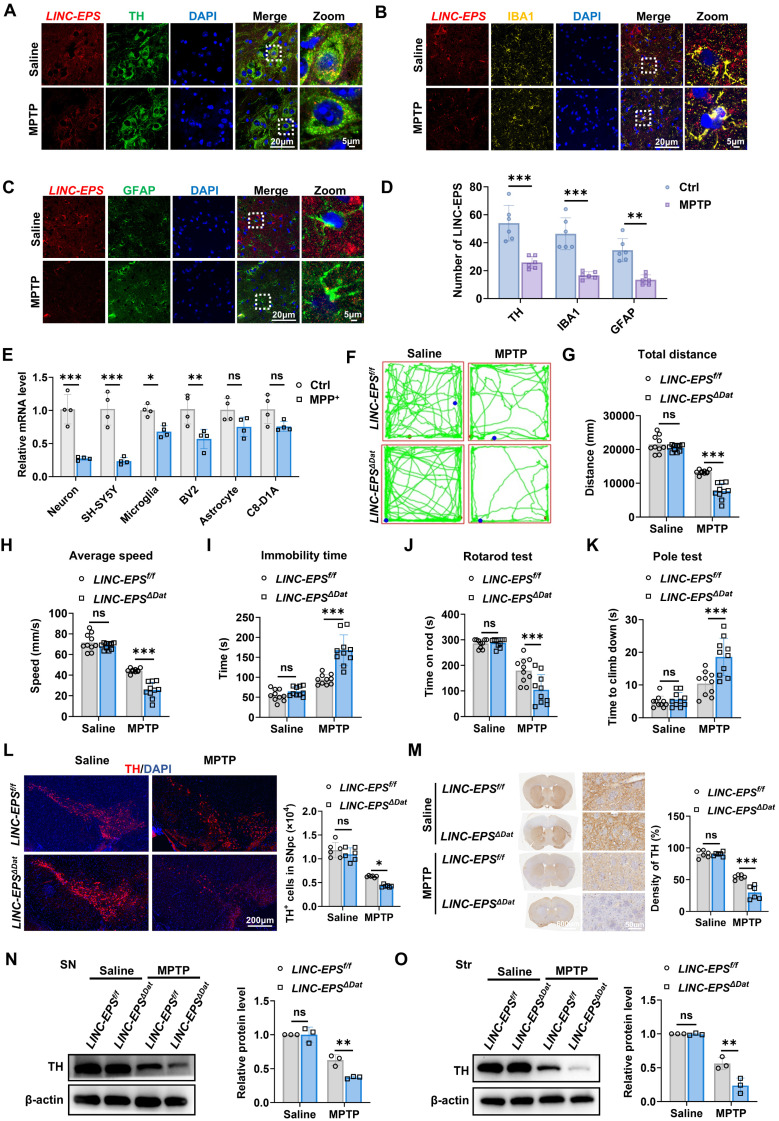
** Loss of LINC-EPS in dopaminergic neurons drives neurodegeneration. (A-C)** Representative RNA-FISH images showing LINC-EPS mRNA puncta (red) co-localized with dopaminergic neurons (TH, green, **A**), microglia (IBA1, yellow, **B**), and astrocytes (GFAP, green, **C**). Nuclei were counterstained with DAPI (blue). Scale bar, 5 µm. **(D)** Quantification of the average number of LINC-EPS mRNA puncta per cell for each cell type, based on the analysis of images as shown in (a-c). n = 6 images per group (3 mice/group; 2 consecutive sections/mouse; 1 predefined field of view/section). **(E)** RT-qPCR analysis of *LINC-EPS* mRNA levels in primary murine neurons, microglia, and astrocytes, alongside the SH-SY5Y (neuronal), BV2 (microglial), and C8-D1A (astrocytic) cell lines, following MPP⁺ exposure (n = 4). **(F-I)** Representative locomotor traces from the open-field test for the indicated groups (*LINC-EPS^f/f^* control mice and *LINC-EPS^ΔDat^* mice with DA neuron-specific LINC-EPS deletion, treated with saline or MPTP) **(F)** and quantification of total distance **(G)**, average speed **(H)**, and immobility time **(I)** (n = 10). Mouse genotype validation is shown in Supplementary Fig. S2C.** (J)** Motor performance on the accelerating rotarod, quantified as latency to fall (n = 10). **(K)** Time to descend in the pole test (n = 10). **(L)** Representative immunofluorescence images and stereological quantification of TH⁺ neurons in the SNpc. Scale bar, 200 µm (n = 6). **(M)** Representative immunohistochemistry images and quantification of TH⁺ fiber density in the striatum. Scale bar, 600 µm (overview) and 50 µm (magnified view) (n = 6).** (N)** Representative immunoblots and quantification of TH protein levels in the SN (n = 3). **(O)** Representative immunoblots and quantification of TH protein levels in the striatum (n = 3). Data are presented as mean ± SEM. Statistical significance was determined by two-way ANOVA with Tukey's post-hoc test. *P < 0.05, **P < 0.01, ***P < 0.001. ns, not significant.

To delineate the cellular specificity of LINC-EPS downregulation in PD brain, we performed RNA fluorescence *in situ* hybridization (RNA-FISH) assay in the SNpc of MPTP-treated mice. This *in vivo* analysis revealed significant reduction in *LINC-EPS* mRNA puncta in DA neurons (TH⁺), microglia (IBA1⁺), and astrocytes (GFAP⁺) following MPTP administration (Fig.2 A-D). To dissect the cell-autonomous responses to neurotoxic insult, we exposed primary murine neural cells and corresponding cell lines to MPP⁺. In stark contrast to the generalized *in vivo* responses, RT-qPCR analysis revealed that the MPP⁺-induced suppression of LINC-EPS was substantially more pronounced in DA neuronal cells than in glial cells (Fig. 2E). To determine the potential effects of neuron* LINC-EPS* on motor deficits and nigrostriatal neurodegeneration in PD model mice, we generated mice with DA neuron-specific deletion of LINC-EPS (*LINC-EPS^f/f^*; Dat-Cre, hereafter referred to as *LINC-EPS^ΔDat^*) and subjected them to MPTP neurotoxicity tests. Successful generation and validation of *LINC-EPS^ΔDat^* mice were confirmed by genotyping PCR (Fig. S2B). Subsequent behavioral assessments revealed that *LINC-EPS^ΔDat^* mice exhibited significantly exacerbated motor deficits compared to their control (*LINC-EPS^f/f^*) littermates. These included impairments in locomotor activity (open-field test; Fig. 2F-I) and motor coordination (rotarod test; Fig. 2J) and increased bradykinesia (pole test; Fig. 2K).

Stereological counting indicated that MPTP-induced loss of TH⁺ neurons in SNpc was significantly greater in *LINC-EPS^ΔDat^* mice than in controls (Fig. 2L). This was paralleled by a more pronounced reduction in the density of TH⁺ nerve terminals in the striatum (Fig. 2M). Immunoblotting analysis further corroborated these findings, showing a greater reduction in TH protein levels in the SN and striatum of *LINC-EPS^ΔDat^* mice than in the SN and striatum of controls (Fig. 2N and O).

Collectively, these results suggest that DA neuron-specific deficiency of *LINC-EPS* aggravates nigrostriatal neurodegeneration and motor deficits in MPTP-induced PD model mice.

### LINC-EPS deficiency promotes ferroptosis in DA neurons

**Figure 3:**
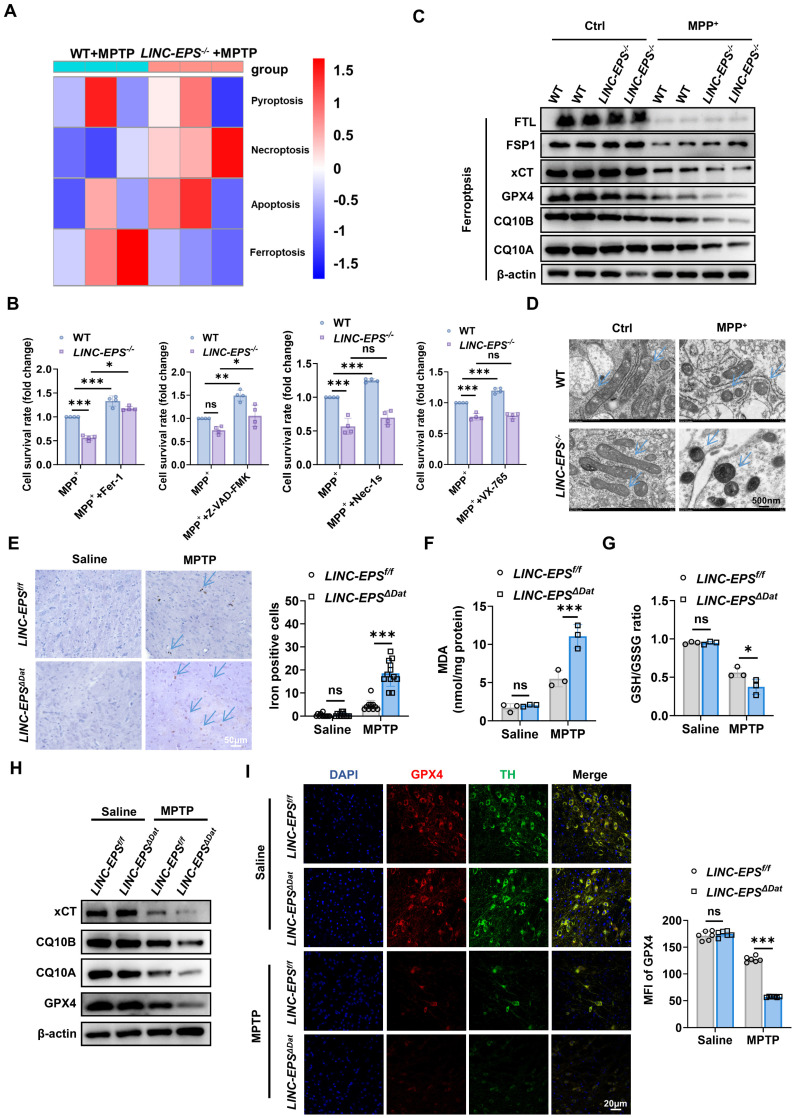
***LINC-EPS* deficiency Promotes Ferroptosis in Dopaminergic Neurons *In vitro* and *In vivo*. (A)** Heatmap of differentially expressed genes from regulated cell death pathways, identified by RNA-seq of SN tissue from MPTP-treated WT and *LINC-EPS^⁻/⁻^* mice. **(B)**Relative cell viability of primary WT and LINC-EPS⁻/⁻ neurons treated with MPP⁺ in the presence of inhibitors for ferroptosis (Ferrostatin-1, Fer-1), apoptosis (Z-VAD-FMK), necroptosis (Necrostatin-1s, Nec-1s), or pyroptosis (VX-765). Cell viability of MPP⁺-treated WT neurons was set as 1.0 to assess the protective effects of inhibitors (n = 4). **(C)** Representative immunoblots of ferroptosis-related proteins (FTL, FSP1, xCT, GPX4, CQ10B, CQ10A) in primary neurons from WT and *LINC-EPS^⁻/⁻^* mice treated with or without MPP⁺ (500 μM, 24 h). Due to limited primary neuron yield, two technical replicates from pooled neurons (six mice per pool) **(D)** Representative TEM images of mitochondria in primary neurons from WT and* LINC-EPS^⁻/⁻^* mice treated with vehicle or MPP⁺. Blue arrows indicate ferroptosis-associated mitochondrial alterations, including shrinkage, increased membrane density, and outer membrane rupture. Scale bar, 500 nm. **(E)** Representative images of Prussian blue staining for iron in the SN of *LINC-EPS^f/f^* and *LINC-EPS^ΔDat^* mice treated with saline or MPTP. Right: Quantification of the number of iron-positive cells per microscopic field (n = 12). Blue arrows highlight iron-positive cells. Scale bar, 50 µm. **(F, G)** Quantification of MDA content, an end-product of lipid peroxidation** (F)** and GSH/GSSG ratio as an indicator of the cellular redox state** (G)** in SN tissue from MPTP-treated *LINC-EPS^f/f^* and *LINC-EPS^ΔDat^* mice (n = 3). **(H)** Representative immunoblots of ferroptosis-related proteins in SN tissue from *LINC-EPS^f/f^* and *LINC-EPS^ΔDat^* mice treated with MPTP. Quantification is provided in Figure S2C. **(I)** Representative co-immunofluorescence images of GPX4 (red) and TH (green) in the SNpc of *LINC-EPS^f/f^* and *LINC-EPS^ΔDat^* mice treated with MPTP. Right: Quantification of GPX4 MFI measured within the soma of individual TH-positive neurons (n = 6). Scale bar, 20 µm. Data are presented as mean ± SEM. Statistical significance was determined by two-way ANOVA with Tukey's post-hoc test or a two-tailed unpaired Student's t-test. *P < 0.05, **P < 0.01, ***P < 0.001. ns, not significant.

To elucidate the mechanisms by which LINC-EPS deficiency exacerbates nigrostriatal neurodegeneration, we performed RNA sequencing (RNA-seq) of the SN tissues of MPTP-treated WT and *LINC-EPS^⁻/⁻^* mice, given that the SN is enriched in dopaminergic neuron somata and constitutes the primary site of selective vulnerability in Parkinson's disease. This analysis revealed the broad dysregulation of multiple cell death pathways in LINC-EPS-deficient tissues. Notably, although gene sets associated with pro-inflammatory death pathways, such as necroptosis and pyroptosis, were upregulated, ferroptosis-related genes were broadly dysregulated, exhibiting a predominant net reduction in anti-ferroptotic factors (Fig. 3A). To functionally dissect the contribution of these pathways, we treated primary neurons with specific cell death inhibitors concurrently with MPP⁺ (the active toxic metabolite of MPTP, 500 µM) for 24 hours. Pharmacological screening revealed that enhanced cell death in *LINC-EPS^⁻/⁻^* neurons was effectively rescued by the ferroptosis inhibitor, ferrostatin-1 (Fer-1). Partial rescue was also observed with the pan-caspase inhibitor, Z-VAD-FMK, whereas inhibitors of necroptosis (Nec-1s) and pyroptosis (VX-765) conferred no significant protection against exacerbated cell loss (Fig. 3B). To probe the underlying molecular alterations, we examined the key regulators and markers of canonical cell death pathways, including ferroptosis, apoptosis, pyroptosis, and necroptosis, via immunoblotting. *LINC-EPS* deficiency in neurons led to the marked downregulation of core anti-ferroptotic proteins (cystine/glutamate antiporter subunit) xCT and GPX4 as well as the coenzyme Q complex (CQ10B and CQ10A) following MPP^+^ treatment (Fig. 3C). In contrast, expression levels of ferroptosis suppressor protein 1 (FSP1) and ferritin light chain (FTL) were not significantly altered between genotypes after MPP⁺ treatment (Fig. 3C). Moreover, no significant differences were observed in the expression levels of key proteins related to apoptosis, pyroptosis, and necroptosis between LINC-EPS^⁻/⁻^ and WT neurons after MPP⁺ treatment (Fig. S2A). Collectively, these convergent lines of evidence identified dysregulated ferroptosis as the primary mechanism driving heightened neuronal vulnerability associated with LINC-EPS loss.

At the ultrastructural level, transmission electron microscopy (TEM) of MPP⁺-treated *LINC-EPS^⁻/⁻^* primary neurons revealed hallmark features of ferroptosis, including shrunken mitochondria with increased membrane density and ruptured outer membranes (Fig. 3D) 38-40**,** confirming ferroptotic mitochondrial damage at the organellar level.

To ascertain whether *LINC-EPS* deficiency exacerbates ferroptosis *in vivo*, we assessed the SN of MPTP-treated *LINC-EPS*^ΔDat^ mice for key ferroptotic markers. As iron dyshomeostasis is a central feature of ferroptosis, we assessed iron accumulation in this study 41, 42. Prussian blue staining revealed a significant increase in the number of iron-positive cells in the SN of MPTP-challenged LINC-EPS*^ΔDat^* mice compared to that in the SN of controls (Fig. 3E). This finding was corroborated by the core biochemical signatures of ferroptosis in the SN tissue lysates. MPTP-treated LINC-EPS*^ΔDat^* mice exhibited markedly higher levels of the lipid peroxidation marker malondialdehyde (MDA) and significantly lower ratio of reduced to oxidized glutathione (GSH/GSSG; Fig. 3F and G). Immunoblotting analysis of SN lysates revealed significantly lower protein levels of xCT, CQ10B, CQ10A, and GPX4 in MPTP-treated *LINC-EPS*^ΔDat^ mice than in MPTP-treated *LINC-EPS*^f/f^ littermate controls (Figs. 3H and S2C). To determine whether GPX4 reduction occurred within DA neurons, we performed co-immunofluorescence analysis of SNpc. This experiment confirmed that GPX4 protein signals were significantly diminished in the TH-positive neurons of MPTP-challenged LINC-EPS*^ΔDat^* mice (Fig. 3I).

Taken together, these findings showed that DA neuron-specific loss of *LINC-EPS* exacerbated ferroptosis in MPTP-induced PD model mice.

### DA neuron-specific overexpression of LINC-EPS attenuates MPTP-induced nigrostriatal pathology

**Figure 4:**
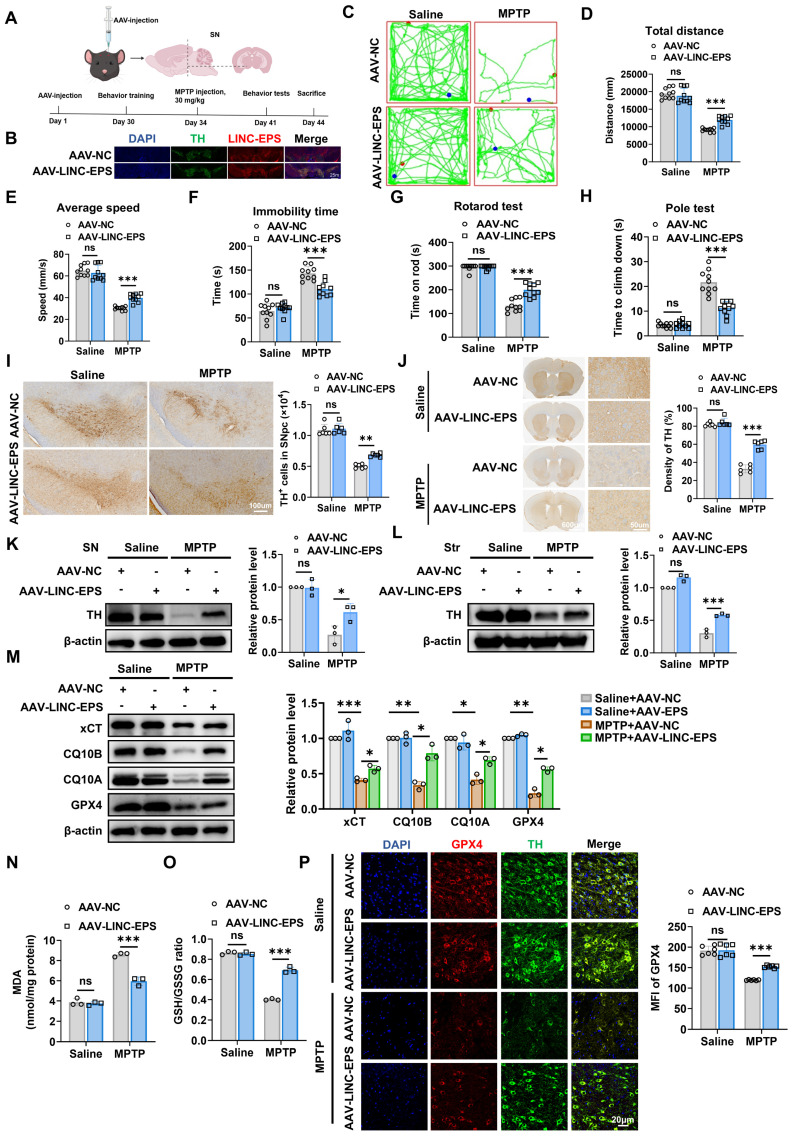
** Overexpression of LINC-EPS in Nigral Dopaminergic Neurons Mitigates MPTP-Induced Nigrostriatal Degeneration and Ferroptosis. (A)** Experimental design: bilateral SNpc injection of AAV-DIO-LINC-EPS or control vector in DAT-Cre mice (3 µL/side; 2×10¹² vg/mL), followed by MPTP administration after 34 days. **(B)** FISH detection of LINC-EPS transcripts (red) combined with TH immunofluorescence (green) in the SN of C57BL/6J mice following injection of AAV-LINC-EPS or AAV-NC. DAPI (blue) labels cell nuclei. Insets show high-magnification views of representative TH-positive neurons co-expressing LINC-EPS. Scale bar = 25m. **(C)** Representative locomotor traces from the open-field test for each group. **(D)** Quantification of the total distance traveled by mice in the open-field test (n = 10). **(E)** Quantification of the average speed of mice during the open-field test (n = 10). **(F)** Quantification of the total immobility time during the open-field test (n = 10). **(G)** Assessment of motor coordination, showing the latency to fall from the accelerating rotarod (n = 10).** (H)** Assessment of bradykinesia, measured as the time required to descend in the pole test (n = 10). **(I)** Representative Immunohistochemistry images of TH positive neurons in the SNpc (n = 6). **(J)** Representative Immunohistochemistry images of TH-positive fibers in the striatum (n = 6). **(K, L)** Representative immunoblots and quantification of TH protein levels in the SN **(L)** and striatum **(L)** (n = 3). **(M)** Ferroptosis-related protein expression (xCT, CQ10B, CQ10A, GPX4) in SN (n=3).** (N, O)** Quantification of MDA levels **(N)** and the GSH/GSSG ratio **(O)** in SN lysates (n = 3). **(P)** GPX4-TH colocalization in SNpc with quantification of GPX4 intensity in TH-positive neurons (scale bar: 20 µm; n=6). Data are presented as mean ± SEM. Statistical significance was determined by two-way ANOVA with Tukey's post-hoc test. *P < 0.05, **P < 0.01, ***P < 0.001. ns, not significant.

After determining that LINC-EPS deficiency exacerbated MPTP-induced nigrostriatal pathology, we examined whether the selective elevation of LINC-EPS in midbrain DA neurons mitigates these degenerative changes. To achieve DA neuron-specific overexpression, DAT^IRES-Cre^ mice were bilaterally injected with a Cre-dependent AAV vector encoding LINC-EPS (AAV-DIO-LINC-EPS) or a corresponding control vector (AAV-DIO-NC) under the hSyn promoter into SNpc using stereotaxic coordinates anteroposterior (AP) -3.0 mm, mediolateral (ML) ±1.3 mm, dorsoventral (DV) -4.4 mm relative to bregma. This strategy selectively drove LINC-EPS expression in Cre-expressing DA neurons. Thirty-four days post-injection, the mice were administered MPTP (30 mg/kg, intraperitoneally, once daily for five consecutive days) or saline. Then, motor performance, nigrostriatal DA integrity, and ferroptosis-associated indices were evaluated (Fig. 4A). FISH combined with TH immunofluorescence revealed that AAV-mediated LINC-EPS delivery resulted in the marked elevation of LINC-EPS transcript signals in TH-positive DA neurons of SN compared to those in the AAV-NC group (Fig. 4B), indicating that the AAV-LINC-EPS construct effectively enhanced LINC-EPS expression in the targeted neuronal populations.

Next, we assessed the effects of the AAV-LINC-EPS construct on motor functions. In the open field test, MPTP treatment caused a marked reduction in locomotor activity in AAV-NC mice, as shown by the representative traces. However, AAV-*LINC-EPS* administration significantly mitigated these motor impairments, as evidenced by their greater total distance traveled, higher average speed, and lower immobility time compared to the MPTP-treated AAV-NC group (Fig. 4C-F). Furthermore, in motor coordination and bradykinesia tests, AAV-*LINC-EPS* group exhibited a significantly longer latency to fall in the rotarod test (Fig. 4G) and shorter descent time in the pole test (Fig. 4H). Consistent with behavioral data, LINC-EPS overexpression robustly protected against MPTP-induced DA neurodegeneration. We observed significant preservation of TH-positive neurons in the SNpc (Fig. 4I) and TH-positive nerve terminals in the striatum (Fig. 4J).

Immunoblotting revealed that the MPTP-induced loss of TH protein was significantly attenuated in both the SN (Fig. 4K) and striatum (Fig. 4L) of AAV-LINC-EPS-administered mice compared to that in the SN and striatum AAV-NC controls. We further examined whether AAV-LINC-EPS treatment attenuates ferroptosis in the MPTP-induced subacute model of PD, as indicated by changes in ferroptosis-associated biochemical and molecular markers. Immunoblotting analysis of SN tissues showed that MPTP treatment caused significant downregulation of the anti-ferroptotic regulators xCT, CQ10B, CQ10A, and GPX4 in AAV-NC mice. However, AAV-*LINC-EPS* delivery significantly counteracted this effect (Fig. 4M). Consistent with these protein-level observations, biochemical analysis of the SN tissues of MPTP-treated mice showed that *AAV-LINC-EPS* markedly suppressed the accumulation of lipid peroxidation marker MDA (Fig. 4N) and prevented the decline in the GSH/GSSG ratio (Fig. 4O). Co-immunofluorescence staining indicated that GPX4 protein levels were significantly higher in the TH-positive neurons of the AAV-*LINC-EPS* group than in those of the AAV-NC group (Fig. 4P).

Collectively, these data demonstrated that AAV-mediated overexpression of *LINC-EPS* in neurons partially protected DA neurons from MPTP-induced degeneration and ferroptosis.

### LINC-EPS negatively regulates ferroptosis in a neuronal cell PD model

**Figure 5:**
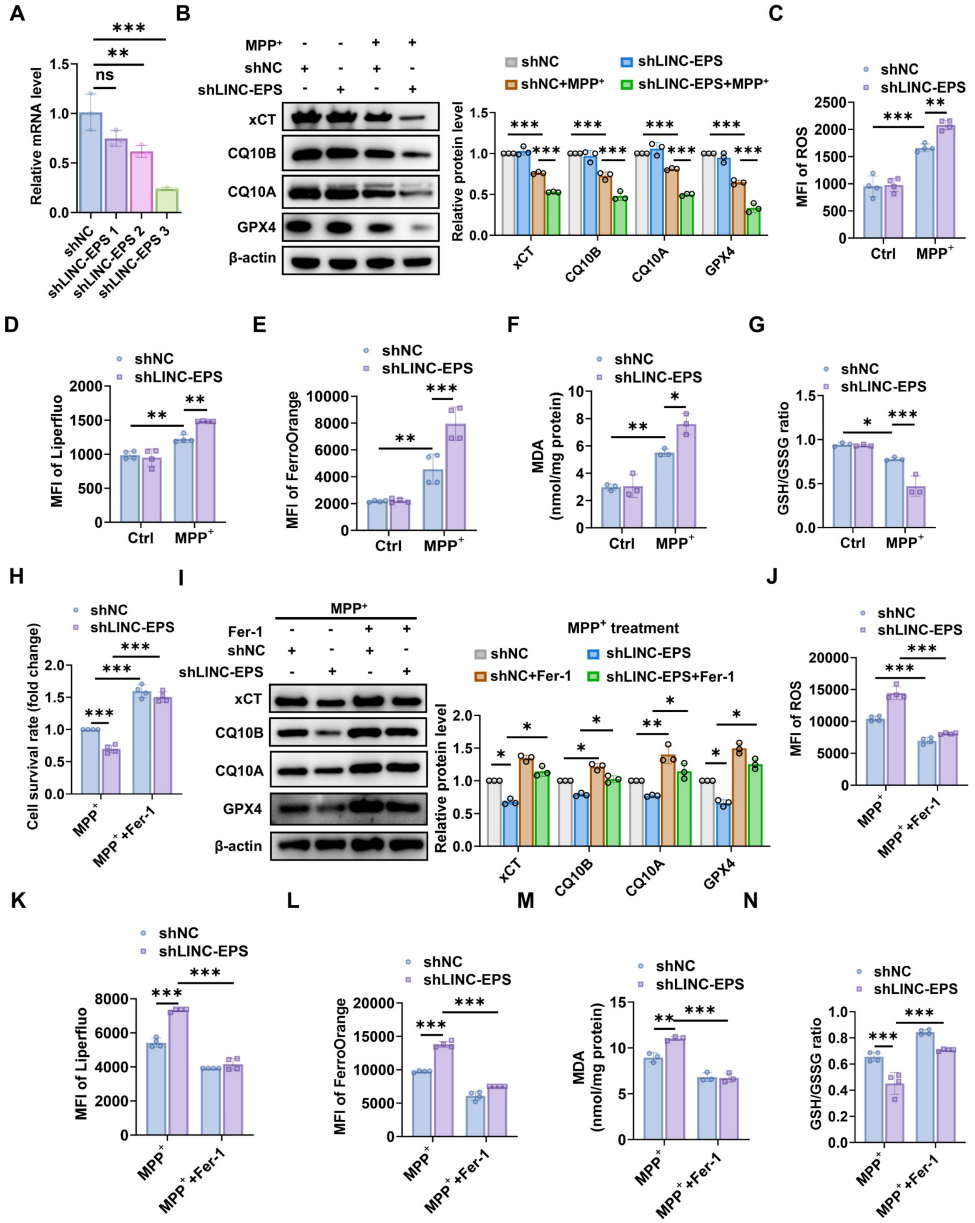
***LINC-EPS* knockdown sensitizes SH-SY5Y cells to MPP⁺-induced ferroptosis.** All experiments were performed in SH-SY5Y cells stably expressing a non-targeting control shRNA (shNC) or an shRNA against LINC-EPS (shLINC-EPS). Cells were treated with or without MPP⁺ (500 µM, 24 h).**(A)** qRT-PCR analysis of LINC-EPS mRNA levels confirming knockdown efficiency (n = 3). **(B)** Immunoblot and corresponding quantification of xCT, CQ10B, CQ10A, and GPX4 protein levels. β-actin served as a loading control (n = 3). **(C)** Quantification of total intracellular reactive oxygen species (ROS) using the fluorescent probe DHE (n = 4). **(D)** Measurement of lipid ROS accumulation using the lipid peroxidation-specific fluorescent probe Liperfluo (n = 4). **(E)** Determination of Fe²⁺ levels using the fluorescent probe FerroOrange (n = 4). **(F)** Quantification of MDA levels as an index of lipid peroxidation (n = 3). **(G)** Determination of the GSH/GSSG ratio as a marker of cellular redox balance (n = 3). **(H-N)** shLINC-EPS and shNC cells were challenged with MPP⁺ in the presence or absence of the ferroptosis inhibitor ferrostatin-1 (Fer-1; 1 µM) for 24 h. **(H)** Cell viability assessment using the CCK-8 assay (n = 4). **(I)** Representative immunoblots and corresponding quantitative analysis of the anti-ferroptosis-related proteins xCT, CQ10B, CQ10A, and GPX4, with β-actin as the loading control (n = 3). **(J)** Quantification of total cellular ROS levels using a fluorescent ROS probe (n = 4). **(K)** Quantification of lipid ROS levels using the Liperfluo probe (n = 4). **(L)** Quantification of Fe²⁺ levels using the FerroOrange probe (n = 4). **(M)** Quantification of MDA levels (n = 3). **(N)** Quantification of the GSH/GSSG ratio (n = 4). Data are presented as mean ± SEM. Statistical significance was determined by a one-way ANOVA followed by Tukey's post-hoc test (A) or two-way ANOVA followed by Tukey's post-hoc test (B-N). *P < 0.05, **P < 0.01, ***P < 0.001.

To elucidate the role of *LINC-EPS* in different cellular contexts, we used a loss-of-function strategy in the SH-SY5Y human DA neuroblastoma cell line. Among the three independent shRNA constructs targeting LINC-EPS, construct #3 showed the most robust knockdown efficiency (Fig. 5A). Therefore, this construct was selected to establish a stable LINC-EPS-knockdown (shLINC-EPS) cell line for subsequent experiments. This cell line exhibited a significant reduction in LINC-EPS transcript levels compared to the non-targeting control (shNC) cells. Upon exposure to neurotoxin MPP⁺, control shNC cells exhibited decreased protein levels of anti-ferroptotic regulators xCT, CQ10B, CQ10A, and GPX4. Notably, LINC-EPS knockdown significantly exacerbated MPP⁺-induced downregulation of all four protein levels (Fig. 5B). Consistent with the loss of these key anti-ferroptosis regulators, shLINC-EPS cells exhibited heightened sensitivity to MPP⁺-induced ferroptotic stress. This was evidenced by a more severe accumulation of total ROS, lipid ROS, intracellular ferrous iron (Fe²⁺), and MDA, along with pronounced reduction in the GSH/GSSG ratio, compared to that in the control cells (Fig. 5C-G).

To examine whether the increased vulnerability of shLINC-EPS cells to MPP⁺ was associated with ferroptosis, a pharmacological rescue experiment was conducted using the specific ferroptosis inhibitor ferrostatin-1 (Fer-1). Specifically, shLINC-EPS cells were challenged with MPP⁺ in the presence or absence of Fer-1. Cell viability assays (CCK-8) demonstrated that Fer-1 significantly improved survival of MPP⁺-treated shLINC-EPS cells compared with MPP⁺-treated vehicle controls (Fig. 5H). Consistent with this effect, immunoblot analysis showed that Fer-1 partially restored the levels of the anti-ferroptotic proteins xCT, CQ10B, CQ10A, and GPX4, which were reduced following MPP⁺ exposure (Fig. 5I). Concurrently, Fer-1 treatment reversed the core biochemical hallmarks of ferroptosis, markedly attenuating MPP⁺-induced accumulation of total ROS, lipid ROS, Fe²⁺, and MDA, while restoring the cellular GSH/GSSG ratio (Fig. 5J-N).

In a complementary gain-of-function experiment, we generated SH-SY5Y cells stably overexpressing *LINC-EPS* (oe*LINC-*EPS; Fig. S3A). In stark contrast to *LINC-EPS* knockdown, its overexpression conferred significant protection against MPP⁺-induced toxicity. Oe*LINC-*EPS cells maintained substantially higher levels of xCT, CQ10B, CQ10A, and GPX4 proteins than the control cells (empty) following MPP^+^ challenge (Fig. S3B). Concordantly, *LINC-EPS* overexpression robustly mitigated the MPP⁺-induced increase in total ROS, lipid ROS, Fe²⁺, and MDA levels and prevented the decrease in the GSH/GSSG ratio (Fig. S3C-G). To directly test whether the neuroprotective function of LINC-EPS is contingent upon the suppression of ferroptosis, we challenged oeLINC-EPS cells with ferroptosis inducer erastin. Although LINC-EPS overexpression robustly increased cell survival, this protective effect was significantly attenuated by co-treatment with erastin (Fig. S3H). At the molecular level, erastin inhibited oeLINC-EPS-mediated upregulation of the levels of anti-ferroptotic proteins xCT, CQ10B, CQ10A, and GPX4 (Fig. S3I). Concurrently, erastin treatment partially attenuated the protective effect of LINC-EPS overexpression against oxidative stress, leading to the significant elevation of total ROS, lipid ROS, Fe²⁺, and MDA levels and reduction of the GSH/GSSG ratio (Fig. S3J-N). Taken together, these findings suggest that the cytoprotective capacity of LINC-EPS is largely dependent on its ability to inhibit ferroptosis.

### LINC-EPS deficiency triggers mitochondrial dysfunction and subsequent ROS-dependent ferroptosis

**Figure 6:**
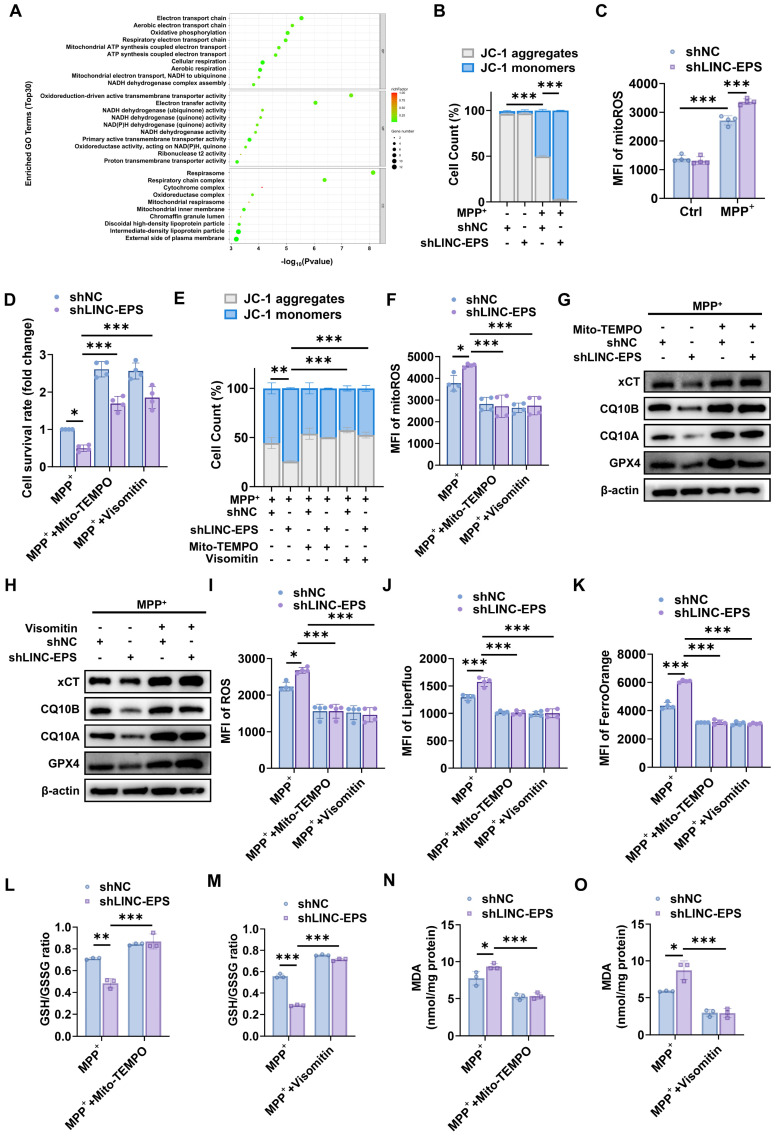
** LINC-EPS deficiency induces mitochondrial dysfunction, leading to ROS-dependent ferroptosis. (A)** Gene Ontology (GO) enrichment analysis of downregulated genes from RNA-seq of SN tissue from MPTP-treated *LINC-EPS*^⁻/⁻^ versus WT mice. The top 30 enriched biological process terms are shown (n = 3). **(B)** Stacked bar chart showing the distribution of JC-1 aggregates (high mitochondrial membrane potential, ΔΨm; gray) and JC-1 monomers (low ΔΨm; blue) in SH-SY5Y cells. Each bar represents one experimental group defined by the combination of MPP⁺ treatment, scrambled control (shNC), or LINC-EPS knockdown (shLINC-EPS), as indicated by the binary notation below ("+", present; "-", absent) and group labels above each bar. Data are presented as percentage of total cell count (n = 4). **(C)** Flow cytometric quantification of mitoROS levels in MPP⁺-treated cells, measured as the MFI of the ROS-sensitive probe MitoSOX (n = 4). **(D-O)** Mitochondrial-targeted antioxidant rescue experiments: cells treated with MPP⁺ (500 µM, 24h) ± Mito-TEMPO (100 µM) or Visomitin (50 nM). **(D)** Quantification of cell survival rate after rescue with Mito-TEMPO or Visomitin (n = 4). **(E)** Quantification of MMP after rescue with Mito-TEMPO or Visomitin (n = 4). **(F)** Quantification of mitoROS levels after rescue with Mito-TEMPO or Visomitin (n = 4). **(G)** Representative immunoblot of xCT, CQ10B, CQ10A, GPX4 after Mito-TEMPO rescue, quantified in Figure S4C. **(H)** Representative immunoblot of xCT, CQ10B, CQ10A, GPX4 after Visomitin rescue, quantified in Figure S4D. **(I)** Quantification of total cellular ROS levels after rescue with Mito-TEMPO or Visomitin (n = 4). **(J)** Quantification of lipid ROS levels after rescue with Mito-TEMPO or Visomitin using the Liperfluo probe (n = 4). **(K)** Quantification of Fe²⁺ levels after rescue with Mito-TEMPO or Visomitin using the FerroOrange probe (n = 4). **(L)** Quantification of the GSH/GSSG ratio after rescue with Mito-TEMPO (n = 3). **(M)** Quantification of the GSH/GSSG ratio after rescue with Visomitin (n = 3). **(N)** Quantification of MDA levels after rescue with Mito-TEMPO (n = 3). **(O)** Quantification of MDA levels after rescue with Visomitin (n = 3). Data are presented as mean ± SEM. Statistical significance was determined by two-way ANOVA with Tukey's post-hoc test. *P < 0.05, **P < 0.01, ***P < 0.001.

To determine the mechanisms by which *LINC-EPS* regulates ferroptosis, we analyzed the RNA-seq data of the SN tissues of MPTP-treated WT and *LINC-EPS^⁻/⁻^* mice. Gene Ontology (GO) enrichment analysis revealed that terms such as “electron transport chain” and “oxidative phosphorylation” were among the most significantly enriched biological processes (Fig. 6A). These data strongly suggest that mitochondrial dysfunction is the primary pathological consequence of LINC-EPS deficiency. Mitochondrial membrane potential (MMP) was assessed in shLINC-EPS cells following MPP^+^ treatment. Upon MPP^+^ exposure, shLINC-EPS cells exhibited a significantly more pronounced decrease in MMP, as evidenced by the reduced percentage of cells containing JC-1 aggregates (high potential), compared to shNC cells (Fig. 6B). Decrease in MMP promoted the production of mitoROS. Accordingly, we observed a marked elevation in mitoROS levels in MPP⁺-treated shLINC-EPS cells relative to the controls (Fig. 6C). Relative to the control cells, oeLINC-EPS cells consistently exhibited preserved mitochondrial membrane potential and reduced mitoROS production following MPP^+^ exposure (Fig. S4A and B). As excessive mitoROS drives ferroptosis 43, 44, we explored whether ROS scavenging alleviates ferroptosis. We treated MPP⁺-challenged shLINC-EPS cells with TEMPO, a general ROS scavenger, or Visomitin, a mitochondria-targeted antioxidant. Reduced viability of shLINC-EPS cells was significantly rescued by both Mito-TEMPO and Visomitin (Fig. 6D), indicating mitochondrial oxidative stress as the primary driver of cell death. Furthermore, both Mito-TEMPO and Visomitin significantly ameliorated MPP^+^-induced MMP collapse (Fig. 6E) and attenuated mitoROS surge (Fig. 6F).

We investigated whether ROS suppression prevents ferroptosis. Immunoblotting analysis revealed that treatment with Mito-TEMPO (Figs. 6G and S4C) and Visomitin (Figs. 6H and S4D) restored the expression levels of the core ferroptotic defense proteins, xCT, CQ10B, CQ10A and GPX4, which were downregulated by MPP⁺ in shLINC-EPS cells. Both Mito-TEMPO and Visomitin significantly suppressed MPP⁺-induced elevation of total cellular ROS (Fig. 6I), lipid ROS (Fig. 6J), and Fe²⁺ (Fig. 6K) levels. Concurrently, treatment with Mito-TEMPO and Visomitin promoted the recovery of the GSH/GSSG ratio (Fig. 6L and M) and suppressed the accumulation of MDA, a key lipid peroxidation marker (Fig. 6N and O).

Collectively, these findings suggest that LINC-EPS deficiency drives the overproduction of mitochondria-derived ROS, a key proximal event to ferroptosis.

### LINC-EPS sustains the PGC-1α signaling axis to preserve mitochondrial homeostasis and prevent ferroptosis

**Figure 7:**
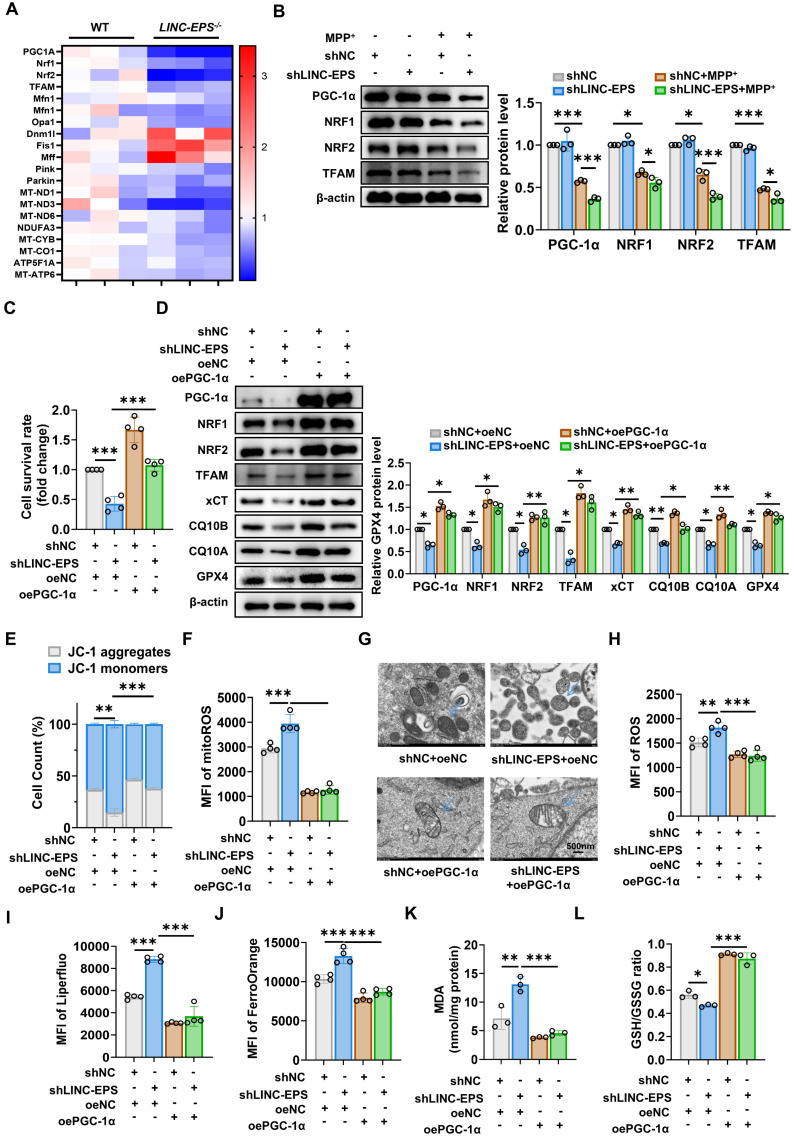
** PGC-1α Restoration Rescues LINC-EPS-Deficient Cells from Mitochondrial Dysfunction and Ferroptosis under MPP⁺-Induced Stress. (A)** Mitochondrial regulatory gene expression profile in primary neurons from WT and LINC-EPS^⁻/⁻^ mice following MPP⁺ exposure (n=3). **(B)** shNC or shLINC-EPS cells were treated ± MPP⁺ (500 µM, 24 h). Representative immunoblots and quantification of PGC-1α, NRF1, NRF2, and TFAM protein levels are shown. β-actin was used as a loading control (n = 3).** (C)** CCK-8 assay assessing cell viability in MPP⁺-treated cells after LINC-EPS knockdown, with or without concurrent PGC-1α overexpression (n = 4). **(D)** Western blot analysis of PGC-1α and its downstream targets in MPP^+^-treated cells with LINC-EPS knockdown and/or PGC-1α overexpression. All groups received MPP^+^ treatment (n = 3).** (E)** Flow cytometric assessment of MMP in MPP⁺-treated shLINC-EPS cells with or without oePGC-1α overexpression, detected using JC-1 dye (n = 4). **(F)** Flow cytometric quantification of mitoROS levels in MPP⁺-treated shLINC-EPS cells with or without PGC-1α overexpression, measured as the MFI using the MitoSOX Red (n = 4). **(G)** Mitochondrial ultrastructure by TEM (scale bar: 500 nm). **(H)** Quantification of total intracellular ROS levels in MPP⁺-treated shLINC-EPS cells with or without PGC-1α overexpression, detected using a general ROS detection probe (n = 4). **(I)** Quantification of lipid ROS levels in MPP⁺-treated shLINC-EPS cells with or without PGC-1α overexpression, measured using the Liperfluo (n = 4). **(J)** Quantification of Fe²⁺ levels in MPP⁺-treated shLINC-EPS cells with or without PGC-1α overexpression, assessed using FerroOrange staining (n = 4). **(K)** Quantification of MDA content as an indicator of lipid peroxidation in MPP⁺-treated shLINC-EPS cells with or without PGC-1α overexpression (n = 3). **(L)** Assessment of GSH/GSSG ratio in MPP⁺-treated shLINC-EPS cells with or without PGC-1α overexpression (n = 3). Data are presented as mean ± SEM. Statistical significance was determined using two-way ANOVA for** (B, D, E)** and one-way ANOVA for **(C, F, H-L)**, with all analyses followed by Tukey's post-hoc test. *P < 0.05, **P < 0.01, ***P < 0.001.

After establishing that LINC-EPS deficiency induces mitochondrial dysfunction, we aimed to identify the underlying molecular drivers. First, we screened the key mitochondrial regulators in primary neurons following MPP⁺-induced stress. This screening revealed significantly downregulated levels of *PGC1A*, which encodes the master transcriptional co-activator PGC-1α (Fig. 7A). As PGC-1α orchestrates mitochondrial biogenesis and antioxidant defense by regulating the activity of transcription factors, such as NRF1, NRF2, and TFAM, we hypothesized that LINC-EPS deficiency impairs the mitochondrial functions by disrupting the PGC-1α transcriptional program.

To verify this hypothesis, we first confirmed that LINC-EPS is essential to maintain the PGC-1α pathway. Challenged with MPP⁺, shLINC-EPS cells showed markedly lower protein levels of PGC-1α and its downstream transcriptional targets, NRF1, NRF2, and TFAM, than shNC cells (Fig. 7B) 45, 46. In contrast to the exacerbation observed with LINC-EPS knockdown, MPP⁺-associated downregulation of the PGC-1α signaling pathway was alleviated in oeLINC-EPS cells (Fig. S5A).

To determine whether the detrimental phenotypes of LINC-EPS deficiency are attributed to impaired PGC-1α signaling, we performed a series of functional rescue experiments. Reduction of cell viability induced by LINC-EPS knockdown was substantially reversed by the ectopic overexpression of PGC-1α (oePGC-1α; Fig. 7C). Immunoblotting analysis confirmed that, under MPP⁺ challenge, this intervention not only restored PGC-1α levels, but also rescued the expression levels of its downstream targets (NRF1, NRF2, and TFAM) and core anti-ferroptotic proteins (xCT, CQ10B, CQ10A and GPX4; Fig. 7D). Moreover, PGC-1α overexpression effectively restored the MMP (Fig. 7E) and suppressed the excessive production of mitoROS (Fig. 7F) in MPP⁺-treated LINC-EPS knockdown cells. TEM provided key morphological evidence: Mitochondria in shLINC-EPS cells exhibited the classic hallmarks of ferroptosis, including reduced size, increased membrane density, and loss of cristae (Fig. 7G). These abnormalities were robustly reversed in the oePGC-1α rescue group.

Crucially, PGC-1α restoration was sufficient to prevent ferroptosis. This was evidenced by the significant reduction in total ROS (Fig. 7H), lipid ROS (Fig. 7I), Fe²⁺ (Fig. 7J), and lipid peroxidation product MDA (Fig. 7K) levels. Remarkably, this neuroprotective treatment not only reversed the oxidative imbalance but also elevated the GSH/GSSG ratio to a level significantly surpassing that in the controls (Fig. 7L). Consistent with this genetic rescue, pharmacological activation of PGC-1α with agonist ZLN005 also significantly mitigated MPP⁺-induced cell death in LINC-EPS-deficient neurons (Fig. S5B). ZLN005 treatment increased the protein levels of PGC-1α, its downstream targets, and ferroptosis defense proteins (Fig. S5C). This activation preserved MMP (Fig. S5D), decreased mitoROS levels (Fig. S5E), and attenuated all measured hallmarks of ferroptosis (Fig. S5F-I) and promoted the recovery of the GSH/GSSG ratio (Fig. S5J).

Collectively, these data suggest that LINC-EPS deficiency drives mitochondrial dysfunction and subsequent ferroptosis by dismantling the PGC-1α transcriptional axis.

### LINC-EPS functions as a transcriptional co-activator by scaffolding PGC-1α to the PGC1A promoter

**Figure 8:**
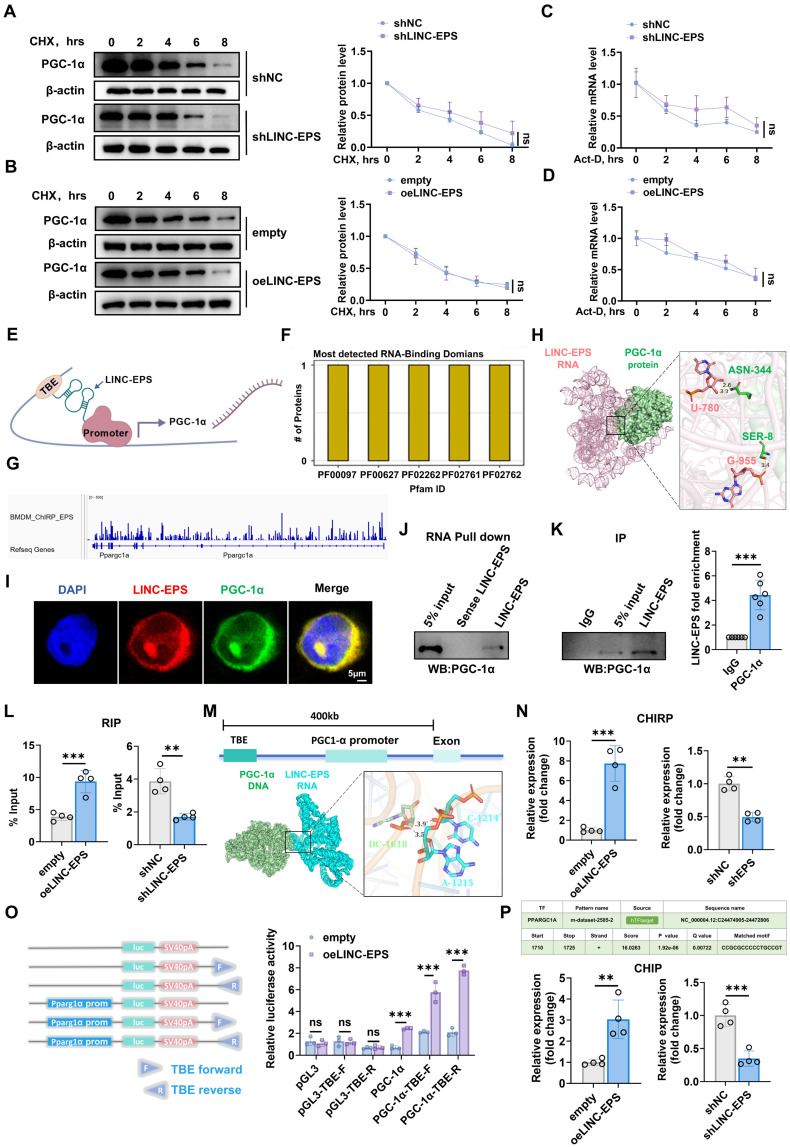
**LINC-EPS Scaffolds PGC-1α for PGC1A Transcriptional Activation. (A-B)** PGC-1α protein stability analysis following cycloheximide (CHX, 50 μg/mL) treatment in LINC-EPS knockdown** (A)** or overexpression** (B)** cells (n=3). **(C-D)** PGC1A mRNA decay kinetics following actinomycin D (5 μg/mL) treatment (n=3). **(E)** Proposed molecular mechanism: LINC-EPS recruits PGC-1α to the TBE regulatory element. **(F)** RNA-binding domain analysis of PGC-1α-LINC-EPS interaction (Pfam database). **(G)** ChIRP-Seq mapping of LINC-EPS occupancy at the PGC1A locus.** (H)** Molecular docking reveals hydrogen bonds between LINC-EPS (U780, G955) and PGC-1α (ASN344, SER8). **(I)** Colocalization of LINC-EPS (red) and PGC-1α (green) by FISH-immunofluorescence (scale bar: 5 μm). **(J)** RNA pull-down validation of LINC-EPS-PGC-1α interaction. **(K)** RIP-qPCR quantification of LINC-EPS enrichment by PGC-1α antibody (n=6). **(L)** LINC-EPS abundance-dependent PGC-1α binding (n=4). **(M)** Predicted RNA-DNA triplex formation between LINC-EPS and TBE element. **(N)** ChIRP-qPCR of LINC-EPS occupancy at TBE (n=4). **(O)** TBE-dependent luciferase reporter activation by LINC-EPS (n=3). **(P)** ChIP-qPCR demonstrating PGC-1α recruitment to PGC1A promoter (+1710-1725; CCGGCCCCCTGCCGT) modulated by LINC-EPS levels (n=4). Data are presented as mean ± SEM. Two-way ANOVA **(A-D)** or Student's t-test **(K-P)**. **P<0.01, ***P<0.001.

To elucidate the mechanism by which LINC-EPS regulates *PGC1A* expression, we assessed its potential role in post-transcriptional regulation. Alterations in LINC-EPS abundance in SH-SY5Y cells did not influence the degradation kinetics of PGC-1α protein, as determined by cycloheximide (CHX) chase assays under both shLINC-EPS and oeLINC-EPS conditions (Fig. 8A and B). Similarly, *PGC1A* mRNA stability, measured after actinomycin D (Act-D) treatment, remained unchanged following modulation of LINC-EPS levels (Fig. 8C and D). These observations suggest that LINC-EPS acts primarily at the transcriptional level to regulate *PGC1A*.

PGC-1α participates in a positive transcriptional feedback loop. LncRNA *Tug1* facilitates PGC-1α protein binding to the *PGC1A* promoter via interaction with a T-box element (TBE) upstream of the gene. Based on this, we hypothesized that LINC-EPS functions as a scaffold to recruit PGC-1α protein to the TBE upstream of the *PGC1A* promoter, thereby enhancing its transcription (Fig. 8E, created with BioGDP.com) 47-49.

Bioinformatics analysis revealed several RNA-binding domains within the PGC-1α protein (Fig. 8F). To investigate whether LINC-EPS directly associates with the chromatin of its target gene, a publicly available Chromatin Isolation by RNA Purification sequencing (ChIRP-Seq) dataset (GSE115524, generated in mouse bone-marrow-derived macrophages) was analyzed 50. The results demonstrated significant LINC-EPS enrichment at the PGC1A genomic locus (Fig. 8G), characterized by distinct peaks in both promoter-upstream and intragenic regions. The upstream peaks were consistent with potential cis-regulatory element interactions, whereas the intronic enrichment suggested involvement in chromatin-associated transcriptional modulation. The genomic distribution of these peaks was concordant with RefSeq annotation, supporting a cis-acting mechanism by which LINC-EPS may influence PGC-1α transcription. Molecular docking predicted direct RNA-protein contact, including hydrogen bonds between U-780 of LINC-EPS and ASN-344 of PGC-1α and between G-955 of LINC-EPS and SER-8 of PGC-1α (Fig. 8H). These interactions were supported by experimental evidence: RNA FISH for LINC-EPS and immunofluorescence assay for PGC-1α revealed nuclear co-localization in SH-SY5Y cells (Fig. 8I). RNA pull-down assays using biotinylated LINC-EPS transcripts specifically precipitated endogenous PGC-1α protein from nuclear lysates, whereas antisense controls showed no binding (Fig. 8J). RNA immunoprecipitation (RIP) with anti-PGC-1α antibody yielded significant enrichment of LINC-EPS compared to the IgG controls, with enrichment increasing in oeLINC-EPS cells and decreasing in shLINC-EPS cells (Fig. 8K and L).

We further evaluated whether LINC-EPS recruits PGC-1α to the *PGC1A* promoter. Upstream TBE, previously linked to *PGC1A* autoregulation, was predicted to form an RNA-DNA triplex with LINC-EPS (Fig. 8M). ChIRP-qPCR analysis revealed the enrichment of LINC-EPS at a genomic region containing the TBE located upstream of the *PGC1A* promoter, which was enhanced by LINC-EPS overexpression and reduced by its knockdown (Fig. 8N). Luciferase reporter assays showed that co-transfection with LINC-EPS increased the promoter activity of constructs containing the full *PGC1A* promoter or minimal promoter with TBE, but not of the TBE-deleted constructs, indicating a TBE-dependent effect (Fig. 8O). Bioinformatics analysis using hTFtarget identified a high-confidence transcription factor binding element for PGC-1α within the PGC1A promoter region (chr4: 24474905-24472806), positioned at nucleotides +1710 to +1725 relative to the transcription start site, with a matched core sequence of CCGGCCCCCTGCCGT. ChIP-qPCR further demonstrated that PGC-1α occupancy at the *PGC1A* promoter was upregulated in oeLINC-EPS cells and downregulated in shLINC-EPS cells (Fig. 8P) 51.

Together, these results support a model in which LINC-EPS serves as a molecular scaffold, recruiting PGC-1α protein to a specific TBE upstream of the *PGC1A* promoter, thereby amplifying a positive transcriptional feedback loop regulating *PGC1A* expression.

### Pharmacological activation of PGC-1α ameliorates MPTP-induced neurotoxicity in LINC-EPS-deficient mice

**Figure 9:**
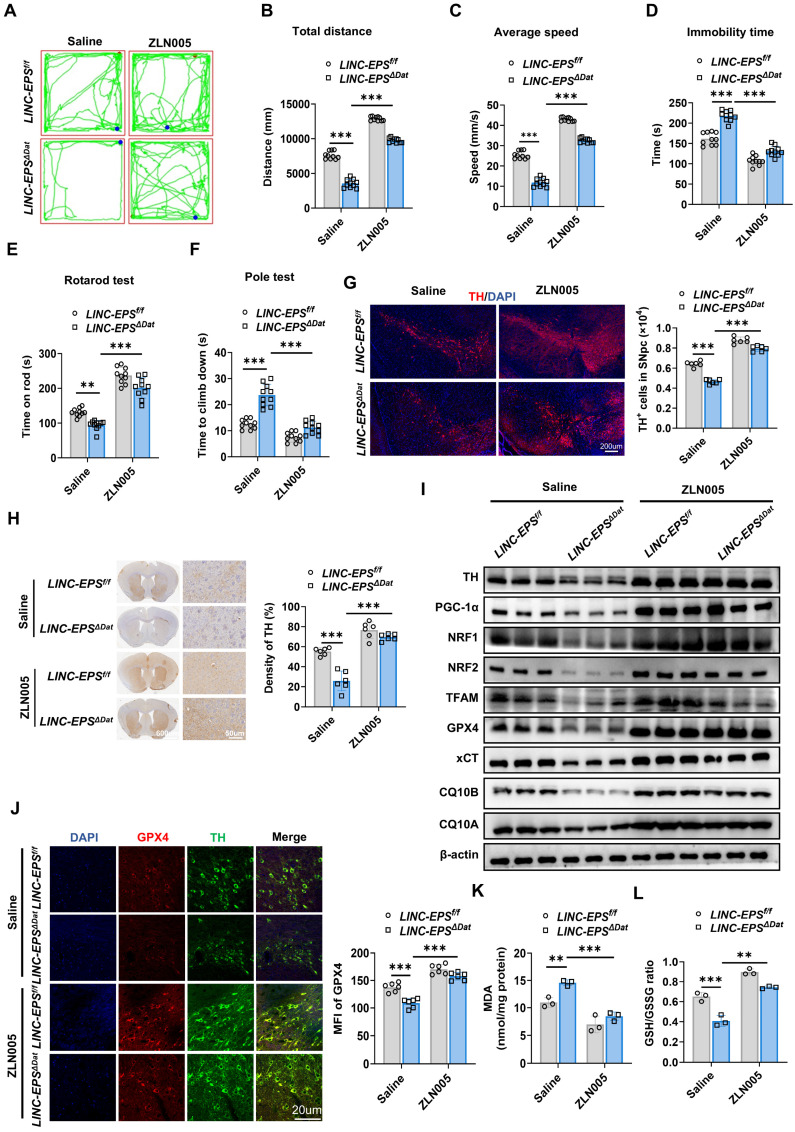
** Pharmacological PGC-1α Activation Protects Against Neurodegeneration in LINC-EPS Knockout PD Model Mice.** Following MPTP intoxication (30 mg/kg, i.p., daily for 5 days), mice of each genotype (*LINC-EPS^f/f^* and *LINC-EPS^ΔDat^*) were subsequently randomly assigned to treatment groups receiving daily administrations of either saline or the PGC-1α agonist ZLN005 (10 mg/kg, i.p.) for a duration of 14 days. Behavioral and pathological analyses were performed 7 days after the last MPTP injection.** (A-D)** Open field test. Representative locomotor traces **(A)** and quantification of total distance traveled** (B)**, average speed** (C)**, and immobility time **(D)** (n = 10). **(E)** Rotarod test, showing latency to fall (n = 10). **(F)** Pole test, showing time to descend (n = 10). **(G)** Representative immunofluorescence images showing TH⁺ neurons (red) in the SNpc, with corresponding stereological quantification on the right. Nuclei were counterstained with DAPI (blue). Scale bar, 200 µm (n = 6). **(H)** Representative immunohistochemistry images of TH-positive fibers in the striatum, including low-magnification overviews and corresponding high-magnification views. The right panel displays the quantification of TH-immunoreactive fiber density. Scale bars, 600 µm (overview) and 50 µm (magnified view) (n = 6).** (I)** Representative immunoblots of TH, PGC-1α pathway proteins, and ferroptosis-related proteins in midbrain lysates. β-actin was used as a loading control. Quantification is shown in Figure S6. **(J)** Representative immunofluorescence images and quantification of GPX4 (red) and TH (green) co-staining in the SNpc. DAPI (blue) stains nuclei (n = 6). Scale bar, 20 µm. **(K)** Quantification of MDA levels in midbrain tissue (n = 3). **(L)** Quantification of the GSH/GSSG ratio in midbrain tissue (n = 3). Data are presented as mean ± SEM. Statistical significance was determined by one-way ANOVA with Tukey's post-hoc test. *P < 0.05, **P < 0.01, ***P < 0.001.

To determine whether pharmacological activation of PGC-1α rescues the heightened pathological sensitivity caused by LINC-EPS deficiency, we administered the PGC-1α agonist ZLN005 to MPTP-treated *LINC-EPS*^ΔDat^ mice. ZLN005 treatment robustly ameliorated the severe motor deficits in *LINC-EPS^ΔDat^* mice 25, 26. In the open-field test, this was evidenced by the increased total distance traveled and average speed, along with reduced immobility time (Fig. 9A-D). Similarly, ZLN005 administration extended the latency to fall in the rotarod test (Fig. 9E) and shortened the descent time in the pole test (Fig. 9F). Although this intervention did not fully restore the performance of *LINC-EPS*^ΔDat^ mice to the level of their ZLN005-treated *LINC-EPS*^f/f^ counterparts, it markedly closed the deficit gap.

This functional recovery was accompanied by significant neuroprotective effects. ZLN005 treatment markedly attenuated the loss of TH-positive neurons in SNpc (Fig. 9G) and reduction of TH-positive fiber density in the striatum (Fig. 9H) of *LINC-EP*S^ΔDat^ mice. At the molecular level, ZLN005 administration increased the expression levels of TH, PGC-1α, its downstream targets (NRF1, NRF2, and TFAM), and key anti-ferroptotic proteins, including xCT, CQ10B, CQ10A and GPX4, in* LINC-EPS^ΔDat^* mice (Figs. 9I and S6). Furthermore, ZLN005 treatment directly counteracted the molecular signature of ferroptosis. This reversed the decrease in GPX4 levels in the surviving TH-positive neurons (Fig. 9J), suppressed the accumulation of lipid peroxidation product MDA (Fig. 9K), and ameliorated the decrease in the GSH/GSSG ratio (Fig. 9L).

Collectively, these *in vivo* data suggest that pharmacological activation of PGC-1α is associated with the rescue of motor function, protection of DA neurons, restoration of the PGC-1α signaling axis and anti-ferroptosis markers in genetically-sensitized PD model mice.

## Discussion

This study identified a previously unrecognized neuroprotective function of LINC-EPS in PD via suppression of ferroptosis in DA neurons. LINC-EPS served as a transcriptional co-activator of PGC-1α by directly binding to a TBE upstream of its promoter region, thereby enhancing PGC-1α expression. This molecular interaction revealed a LINC-EPS/PGC-1α axis that maintained mitochondrial homeostasis, attenuated mitoROS production, and conferred resistance to ferroptotic cell death. Both LINC-EPS overexpression and pharmacological activation of PGC-1α significantly ameliorated motor deficits and preserved DA neuronal integrity in experimental PD models 52-54. Our findings reveal a novel LINC-EPS/PGC-1α neuroprotective axis and identify LINC-EPS as a potential therapeutic target for PD.

LINC-EPS, originally characterized as a regulator of erythroid differentiation and immune responses 55-57, exhibits broad tissue expression including substantial levels in human brain (GTEx database), suggesting neurological functions. In this study, we identified LINC-EPS as a factor implicated in the pathogenesis of PD, demonstrating its consistent downregulation across multiple disease models and patient samples. LINC-EPS expression was decreased in the SN and striatum of MPTP-intoxicated mice and also reduced in peripheral blood samples of patients with PD. Importantly, peripheral LINC-EPS levels correlated inversely with UPDRS III motor scores, linking lower expression to more severe motor impairment. *In vivo* RNA *in situ* hybridization revealed LINC-EPS downregulation across DA neurons, microglia, and astrocytes in MPTP-treated mouse SN, and *in vitro* studies revealed more pronounced suppression in DA neurons, suggesting neuron-specific susceptibility. Our functional validation using both global and conditional knockout strategies, combined with viral-mediated rescue experiments, established LINC-EPS as critical for DA neuron survival. Both global and neuron-specific LINC-EPS-deficient mice demonstrated cell-autonomous neuroprotective functions, with AAV-mediated LINC-EPS restoration reversing motor deficits and neurodegeneration.

Converging pathological and biochemical evidence, including prominent iron accumulation and lipid peroxidation in the SN of patients with PD, implicates ferroptosis as a contributor to DA neuronal loss 12-14. Cellular defense against ferroptosis relies primarily on the glutathione/GPX4 axis and the coenzyme Q system 15-19. In our models, LINC-EPS deficiency broadly dysregulated ferroptosis-related genes and resulted in a net reduction of anti-ferroptotic factors, including mitochondrial CoQ pathway genes (e.g., CQ10A/CQ10B) and core defenses such as xCT and GPX4. To address iron directly, we quantified intracellular labile Fe²⁺ using FerroOrange and flow cytometric MFI, providing a quantitative readout of bioavailable iron relevant to ferroptotic chemistry. Together with increased lipid peroxidation and GSH depletion, these findings support a coordinated collapse of anti-ferroptotic defenses under LINC-EPS loss. Furthermore, our datasets indicate that LINC-EPS deficiency preferentially engages ferroptosis, with limited changes in canonical markers of apoptosis, necroptosis, and pyroptosis. This pathway preference is plausible for DA neurons, which combine high basal oxidative burden from dopamine metabolism, high iron content in the nigrostriatal system, and PUFA-enriched membranes that are susceptible to lipid peroxidation.

Ferroptosis and mitochondrial dysfunction are tightly coupled in our PD-relevant stress context. MPP⁺-driven Complex I inhibition increases mitoROS and perturbs cellular redox balance, thereby facilitating iron-catalyzed lipid peroxidation and weakening GPX4- and CoQ10-dependent antioxidant defenses. In turn, lipid peroxidation damages mitochondrial membranes and impairs bioenergetic function, amplifying ROS production and establishing a feed-forward vicious cycle. Accordingly, ferroptosis inhibitors (e.g., ferrostatin-1 and liproxstatin-1) are expected to confer neuroprotection in part by preserving mitochondrial integrity: by arresting lipid peroxide propagation within mitochondrial membranes, these agents may limit membrane damage, maintain mitochondrial membrane potential, and prevent secondary mitochondrial collapse, thereby breaking the vicious cycle of oxidative stress and bioenergetic failure 8, 37, 57.

Our findings demonstrate that LINC-EPS functions as a molecular scaffold, facilitating a positive transcriptional feedback loop by recruiting PGC-1α to its own promoter. This mechanism sustains a transcriptional program that bolsters mitochondrial antioxidant defenses, including the glutathione/GPX4 system and coenzyme Q10 biosynthesis, thereby suppressing lipid peroxidation and conferring resistance to ferroptotic cell death. The observed downregulation of LINC-EPS in both preclinical models and PD patients suggests that disruption of this protective axis contributes to progressive neurodegeneration. Our work delineates a specific upstream regulatory cascade that controls mitochondrial dysfunction and ferroptotic execution.

Our analysis revealed a significant negative correlation between peripheral PBMC LINC-EPS levels and UPDRS-III motor scores in PD patients, indicating lower LINC-EPS expression in blood associates with greater motor impairment. From a translational perspective, peripheral blood LINC-EPS may offer several clinical utilities, including a supplementary diagnostic biomarker, a progression indicator for longitudinal monitoring, and a potential pharmacodynamic marker to assess therapeutic responses. However, several limitations warrant cautious interpretation. First, age and sex were not matched between PD patients and controls at enrollment in this study. Second, most PD patients were receiving dopaminergic therapy, which may alter PBMC genes expression independently of PD pathology. Third, the cohort size (30 PD and 30 controls) limited covariate-adjusted analyses to control for confounders such as disease duration, medication exposure, systemic inflammation, and comorbidities. Fourth, a direct link between peripheral LINC-EPS levels and nigral pathology in living patients remains unvalidated because matched brain tissue is not available. Future studies are warranted to utilize neuroimaging (e.g., DAT-SPECT or neuromelanin-sensitive MRI) as surrogate markers for nigral pathology to definitively establish this blood-brain correlation in humans. Finally, public transcriptomic resources suggest that LINC-EPS dysregulation may also occur in other neurodegenerative disorders (e.g., Alzheimer's disease and amyotrophic lateral sclerosis), underscoring the need for larger, well-matched, multicenter validation to define disease specificity and clinical utility 21-23.

Although we demonstrated efficacy of LINC-EPS overexpression in alleviating PD pathology, clinical translation of lncRNA-based therapies faces significant hurdles, particularly concerning stability and blood-brain barrier delivery. Consequently, pharmacological activation of the downstream PGC-1α axis currently offers a more accessible therapeutic avenue 24-26. Furthermore, MPTP/MPP⁺ models, though widely used, exhibit important limitations: they do not fully recapitulate the chronic, progressive nature of idiopathic PD; they exhibit incomplete α-synuclein pathology, lacking widespread Lewy body formation; and they predominantly capture motor deficits with inadequate modeling of non-motor symptoms such as cognitive decline and autonomic dysfunction. These constraints necessitate cautious extrapolation of findings to human PD 56-58.

Critical mechanistic gaps remain regarding how MPTP/MPP⁺ triggers LINC-EPS downregulation. MPP⁺ accumulates in mitochondria via dopamine transporter (DAT) uptake and inhibits Complex I, generating excessive ROS and impairing ATP production. This mitochondrial dysfunction and oxidative stress may activate stress-responsive transcription factors (e.g., NF-κB, p53) that repress LINC-EPS transcription or recruit epigenetic modifiers (e.g., DNA methyltransferases, histone deacetylases) to silence the LINC-EPS locus. Additionally, dopamine metabolism produces reactive quinones and hydrogen peroxide, triggering ferroptosis-priming conditions that may feedback to suppress LINC-EPS expression as a maladaptive response. MPTP-induced neuroinflammation, characterized by microglial activation and the release of pro-inflammatory cytokines (TNF-α, IL-1β, IL-6), potentially modulates LINC-EPS expression via inflammation-driven transcriptional reprogramming. These hypotheses regarding the regulatory mechanisms of LINC-EPS warrant further investigation.

## Conclusion

Our study establishes the LINC-EPS/PGC-1α axis as a critical regulator of dopaminergic neuron survival by controlling susceptibility to ferroptosis. The disruption of this axis contributes to neurodegeneration in Parkinson's disease. Pharmacological potentiation of this pathway reverses neurodegenerative phenotypes in preclinical models, identifying it as a promising and mechanistically defined target for developing disease-modifying therapies for Parkinson's disease.

## Supplementary Material

Supplementary figures and tables.

## Data Availability

The datasets supporting the conclusions of this article are included within the article and its additional files.
