# Supplementary material for: LINC-EPS Protects Against Neurodegeneration by Driving a PGC-1α-Mediated Anti-Ferroptosis Program in Parkinson's Disease
Source: Int J Biol Sci. 2026 Mar 17;22(7):3367–88. doi: 10.7150/ijbs.128204 (PMC13085879; doi:10.7150/ijbs.128204)
Supplement: Supplementary file 1 — Supplementary figures and tables. [file ijbsv22p3367s1.pdf]

Supplementary Figure Caption

Fig. S1

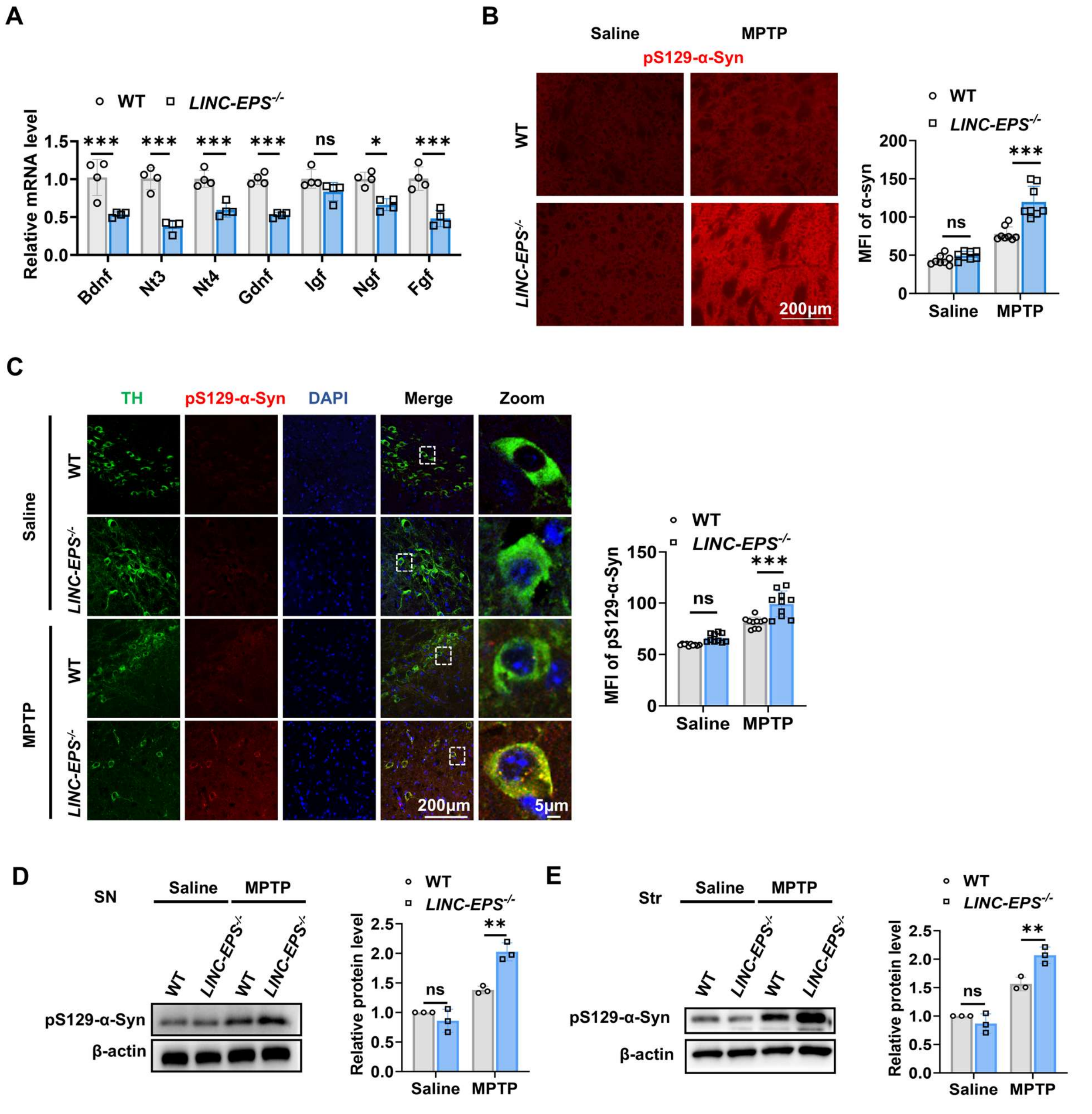

Fig. S1 ***LINC-EPS*** **Deficiency Exacerbates MPTP-Induced Neurochemical and Pathological Alterations**

(A) RT-qPCR analysis of neurotrophic factor mRNA levels (Bdnf, Nt3, Nt4, Gdnf, Igf, Ngf, Fgf) in midbrain tissue from the indicated groups (n = 4). **(B)** Representative immunofluorescence images of pS129-α-Syn (red) in the striatum and quantification of its mean fluorescence intensity (MFI) (n = 8).

Scale bar, 200 μm. **(C)** Representative co-immunofluorescence images for pS129-α-Syn (red), TH (green), and DAPI (blue) in the SNpc. Right panels show magnified views of the boxed areas. Far right: Quantification of pS129-α-Syn MFI within TH^+ neurons (n = 10). Scale bar, 200 μm. **(D, E)** Representative immunoblots and quantification of pS129-α-Syn protein levels in the SN **(D)** and striatum **(E)**. β-actin served as a loading control (n = 3). Data are presented as mean ± SEM. Statistical significance was determined by two-way ANOVA with Tukey's post-hoc test. *$P < 0.05$, **$P < 0.01$, ***$P < 0.001$. ns, not significant.

Fig. S2

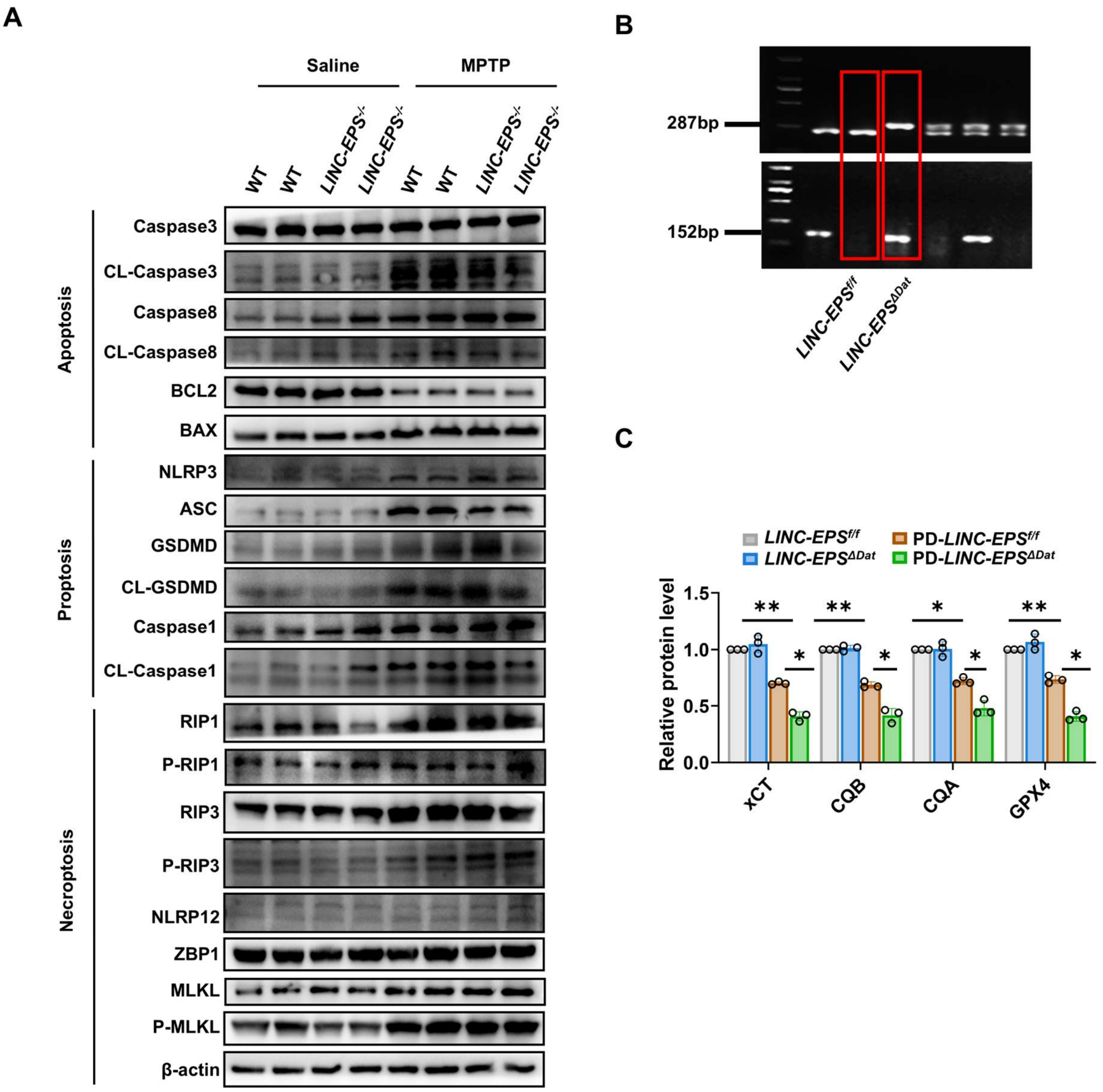

Fig. S2 LINC-EPS Deficiency Does Not Activate Other Major Regulated Cell Death Pathways

(A) Representative immunoblots of key protein markers for apoptosis (CL-Caspase-3), pyroptosis (CL- Caspase-1, GSDMD-N), and necroptosis (P-MLKL, P-RIP3) in primary neurons from WT and *LINC-EPS*$^{-/-}$ mice treated with or without MPP$^+$ (500 μM, 24 h). Due to limited primary neuron yield, two technical replicates from pooled neurons (six mice per pool). (B) Genotyping PCR validation of

dopamine neuron-specific LINC-EPS conditional knockout (*LINC-EPS*$^{\Delta Dat}$) mice. The 287 bp band represents the floxed allele (loxP sites flanking the target region), and the 152 bp band indicates the deleted allele after Cre-mediated excision of the intervening sequence. *LINC-EPS*$^{f/f}$ control mice showed only the 287 bp floxed allele, whereas *LINC-EPS*$^{\Delta Dat}$ mice displayed both the 287 bp band (from non-recombined cells) and the 152 bp band (from dopaminergic neurons with successful Cre-mediated deletion). **(C)** Quantification of relative protein levels for xCT, CQ10B, CQ10A and GPX4 from the immunoblots shown in Figure 3H (n = 3). Data are presented as mean ± SEM. Statistical significance was determined using two-way ANOVA followed by Tukey's post hoc test. *$P < 0.05$, **$P < 0.01$, ***$P < 0.001$; ns, not significant.

Fig. S3

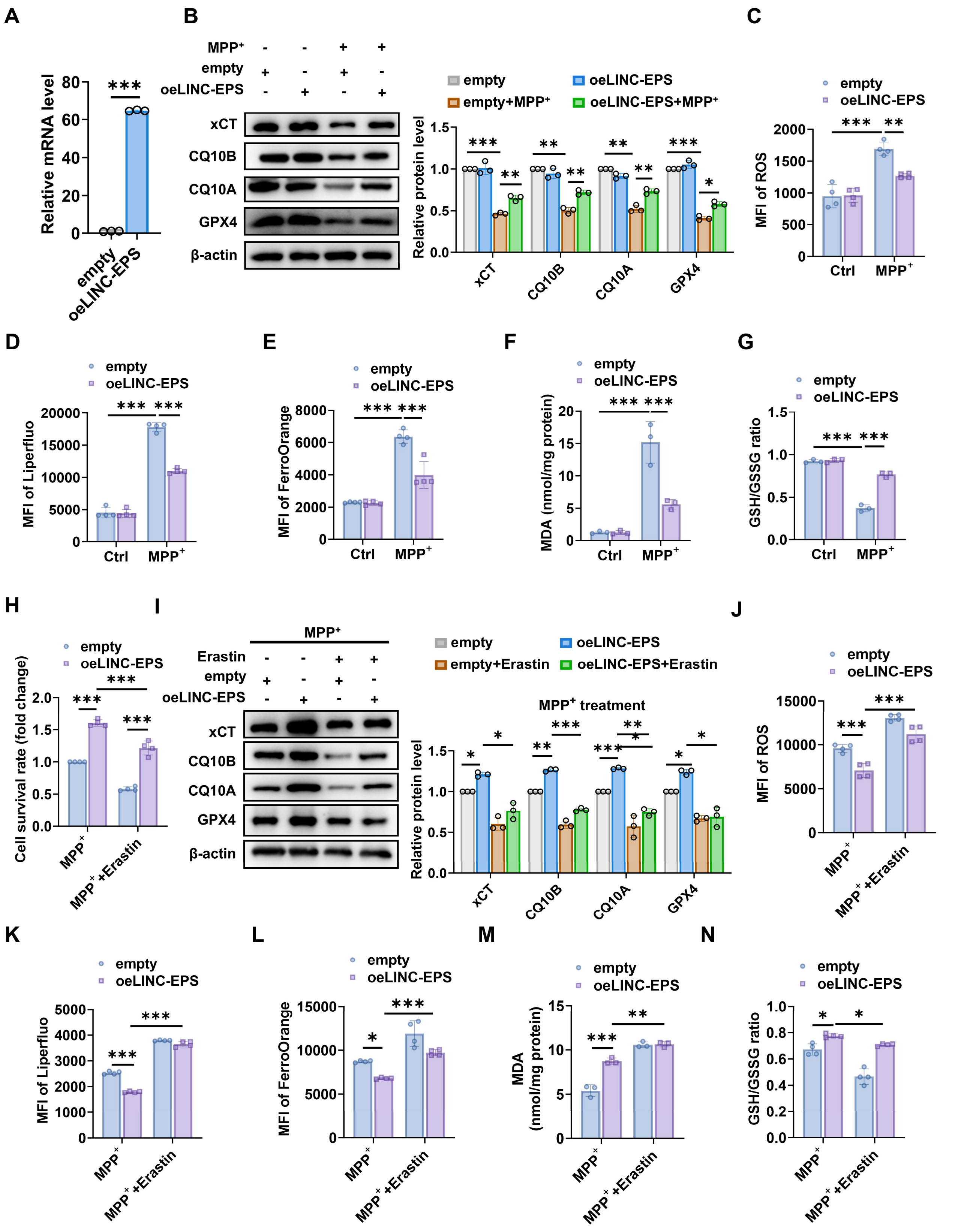
A
B
C
D
E
F
G
H
I
J
K
L
M
N
Relative mRNA level
empty
oeLINC-EPS
MPP⁺
empty
oeLINC-EPS
xCT
CQ10B
CQ10A
GPX4
β-actin
empty
oeLINC-EPS
empty+MPP⁺
oeLINC-EPS+MPP⁺
Relative protein level
MFI of ROS
Ctrl
MPP⁺
MFI of Liperfluo
MFI of FerroOrange
MDA (nmol/mg protein)
GSH/GSSG ratio
Cell survival rate (fold change)
MPP⁺+Erastin
Erastin
empty+Erastin
oeLINC-EPS+Erastin
MPP⁺ treatment
MDA (nmol/mg protein)

Fig. S3 LINC-EPS overexpression protects SH-SY5Y cells from MPP^+- and Erastin-induced ferroptosis

SH-SY5Y cells stably overexpressing LINC-EPS (oeLINC-EPS) or empty vector control were treated with MPP^+ (500 μM, 24 h) with or without erastin (10 μM, 24 h). **(A)** qRT-PCR validation of LINC-EPS overexpression (n = 3). **(B–G)** Effects of LINC-EPS overexpression on MPP^+-induced ferroptosis: **(B)** protein levels of xCT, CQ10B, CQ10A, and GPX4 (n = 3); **(C)** total ROS (n = 4); **(D)** lipid ROS (n = 4); **(E)** Fe^{2+} levels (n = 4); **(F)** MDA content (n = 3); **(G)** GSH/GSSG ratio (n = 3). **(H–N)** Combined effects of LINC-EPS overexpression and erastin treatment: **(H)** cell viability (n = 4); **(I)** xCT, GPX4, CQ10A, and CQ10B protein levels (n = 3); **(J)** total ROS (n = 4); **(K)** lipid ROS (n = 4); **(L)** Fe^{2+} levels (n = 4); **(M)** MDA content (n = 3); **(N)** GSH/GSSG ratio (n = 4). Data are mean ± SEM. Statistical analysis: two-tailed Student's t-test **(A)** or two-way ANOVA with Tukey's post-hoc test **(B–N)**. $^*P < 0.05$, $^{**}P < 0.01$, $^{***}P < 0.001$. Detailed experimental procedures are described in Materials and Methods.

Fig. S4

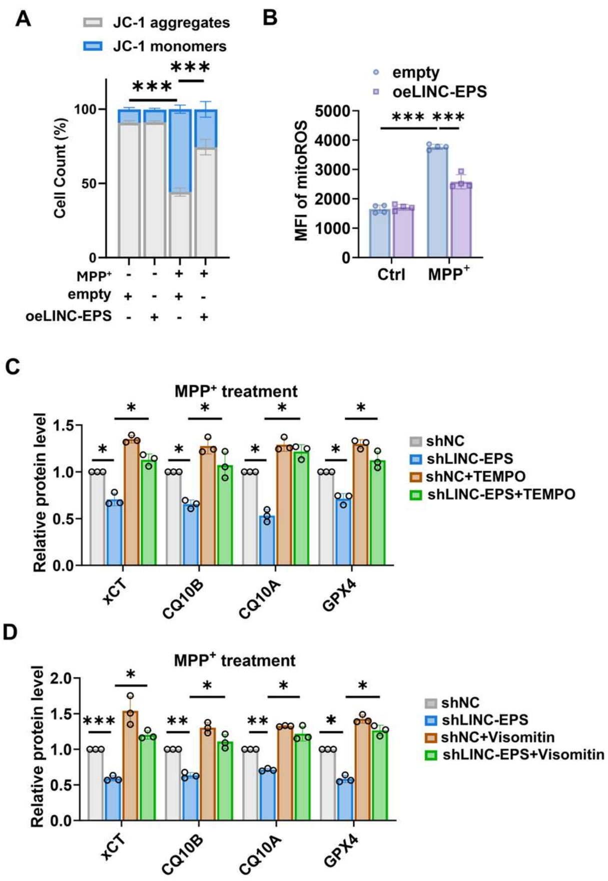

Fig. S4 LINC-EPS Deficiency-Induced Ferroptotic Phenotypes are Rescued by mitoROS Scavengers

(A, B) oeLINC-EPS or empty cells were treated with or without MPP^+ (500 μM, 24 h). **(A)** Flow cytometry analysis of MMP (n = 4). **(B)** Quantification of mitoROS levels (n = 4). **(C)** Quantification of relative protein levels from immunoblots shown in Figure 6G (n = 3). **(D)** Quantification of relative protein levels from immunoblots shown in Figure 6H (n = 3). Data are presented as mean ± SEM. Statistical significance was determined by two-way ANOVA with Tukey's post-hoc test. $^*P < 0.05$, $^{**}P < 0.01$, $^{***}P < 0.001$.

Fig. S5

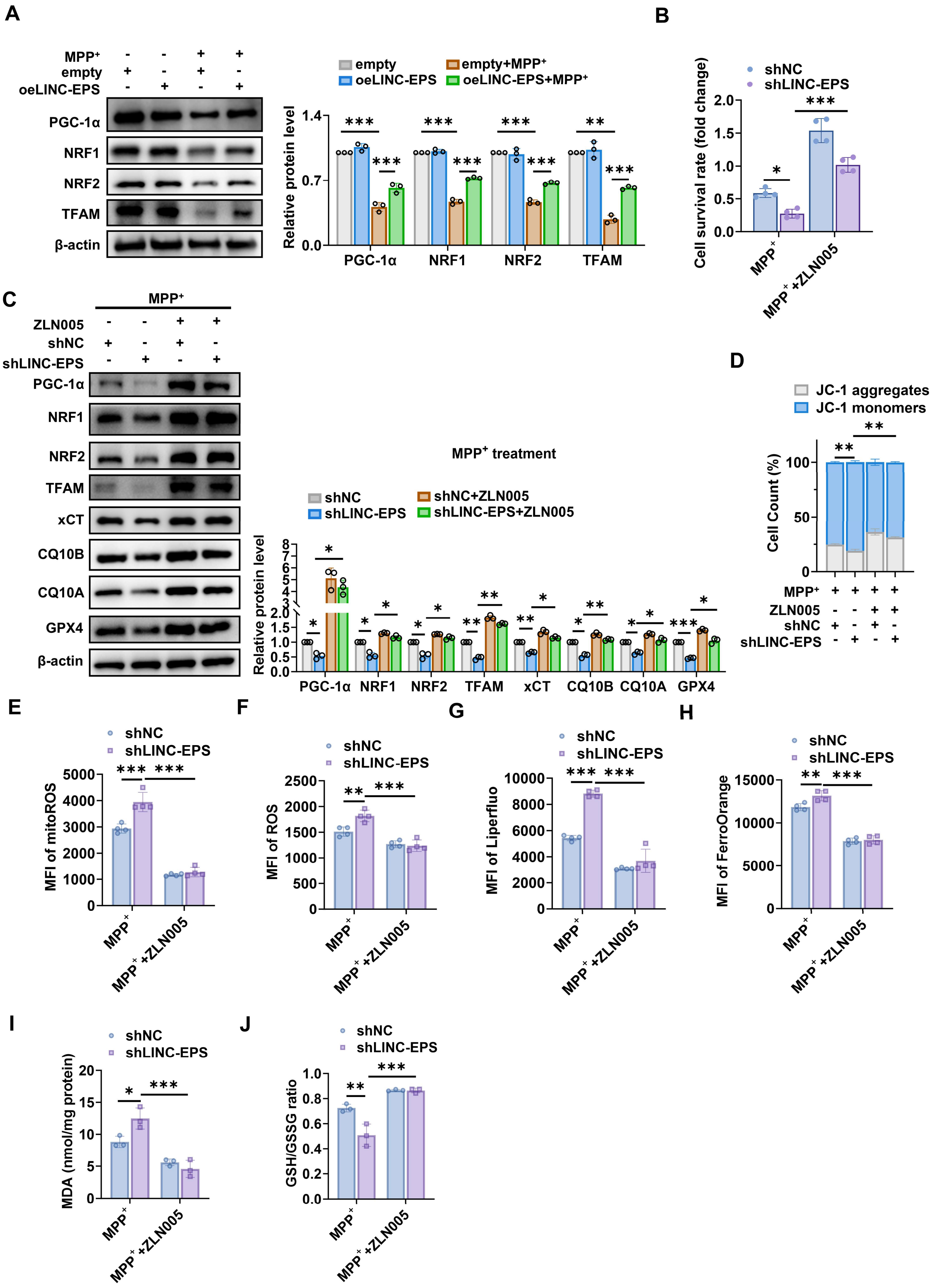
A
MPP+
empty
oeLINC-EPS
PGC-1α
NRF1
NRF2
TFAM
β-actin
empty
empty+MPP+
oeLINC-EPS
oeLINC-EPS+MPP+
Relative protein level
1.4
0.7
0.0
PGC-1α
NRF1
NRF2
TFAM
B
shNC
shLINC-EPS
Cell survival rate (fold change)
2.0
1.5
1.0
0.5
0.0
MPP+
MPP+ +ZLN005
C
MPP+
ZLN005
shNC
shLINC-EPS
PGC-1α
NRF1
NRF2
TFAM
xCT
CQ10B
CQ10A
GPX4
β-actin
MPP+ treatment
shNC
shLINC-EPS
shNC+ZLN005
shLINC-EPS+ZLN005
Relative protein level
PGC-1α
NRF1
NRF2
TFAM
xCT
CQ10B
CQ10A
GPX4
D
JC-1 aggregates
JC-1 monomers
Cell Count (%)
100
50
0
MPP+
ZLN005
shNC
shLINC-EPS
E
shNC
shLINC-EPS
MFI of mitoROS
5000
4000
3000
2000
1000
0
MPP+
MPP+ +ZLN005
F
shNC
shLINC-EPS
MFI of ROS
2500
2000
1500
1000
500
0
MPP+
MPP+ +ZLN005
G
shNC
shLINC-EPS
MFI of Liperfluo
10000
8000
6000
4000
2000
0
MPP+
MPP+ +ZLN005
H
shNC
shLINC-EPS
MFI of FerroOrange
15000
10000
5000
0
MPP+
MPP+ +ZLN005
I
shNC
shLINC-EPS
MDA (nmol/mg protein)
20
15
10
5
0
MPP+
MPP+ +ZLN005
J
shNC
shLINC-EPS
GSH/GSSG ratio
1.0
0.8
0.6
0.4
0.2
0.0
MPP+
MPP+ +ZLN005

Fig. S5 Pharmacological and Genetic Modulation of the LINC-EPS/PGC-1α Axis

(A) PGC-1α axis protein expression in oeLINC-EPS or empty vector cells treated with or without MPP^+ (500 μM, 24 h) (n = 3). **(B–J)** shLINC-EPS cells were treated with MPP^+ (500 μM, 24 h) with or without the PGC-1α agonist ZLN005 (10 μM). **(B)** Cell viability (n = 4); **(C)** PGC-1α axis and ferroptosis defense protein levels (n = 3); **(D)** mitochondrial membrane potential (n = 4); **(E)** mitoROS levels (n = 4); **(F)** total ROS (n = 4); **(G)** lipid ROS (n = 4); **(H)** Fe^{2+} levels (n = 4); **(I)** MDA content (n = 3); **(J)** GSH/GSSG ratio (n = 3). Data are mean ± SEM. Statistical analysis: two-way ANOVA with Tukey's post-hoc test. *P < 0.05, **P < 0.01, ***P < 0.001. Detailed experimental procedures are described in Materials and Methods.

Fig. S6

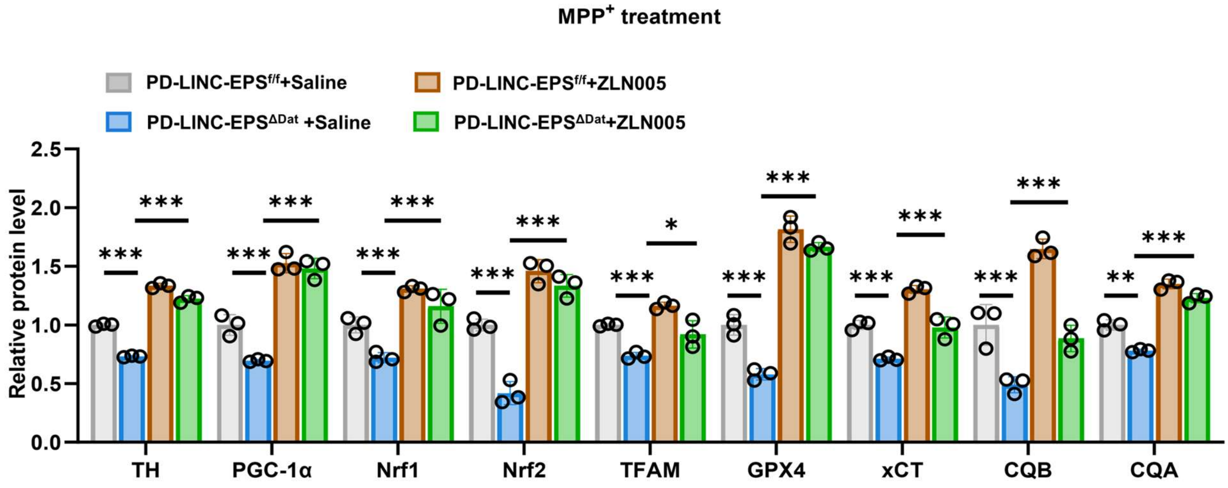

Fig. S6 Quantification of Protein Expression Levels in the Midbrain

Quantification of the immunoblot analysis shown in Figure 9I for TH, PGC-1α, NRF1, NRF2, TFAM, GPX4, xCT, CQ10B, and CQ10A. Protein levels were normalized to β-actin (n = 3). Data are presented as mean ± SEM. Statistical significance was determined by two-way ANOVA with Tukey's post-hoc test. *$P < 0.05$, **$P < 0.01$, ***$P < 0.001$.

Supplementary Materials and Methods

Immunohistochemistry and Imaging

Following terminal anesthesia with Avertin (30 μL/g, i.p.), animals were perfused transcardially with ice-cold saline and subsequently with 4% paraformaldehyde (PFA). Excised brains were post-fixed overnight in 4% PFA at 4°C, followed by cryoprotection in a 30% sucrose solution. Coronal sections (15–30 μm thickness) were then prepared using a Leica cryostat. For immunofluorescence staining, free-floating sections were first permeabilized with 0.3% Triton X-100 and then blocked in 5% BSA. Subsequently, sections were incubated with primary antibodies in the Supplementary Materials (Table S2) overnight at 4°C. After washing, sections were incubated with the appropriate Alexa Fluor-conjugated secondary antibodies and nuclei were counterstained with DAPI. Fluorescence signals were captured on a Zeiss LSM 880 confocal microscope, and subsequent image analysis was performed using Fiji software (ImageJ).

Western Blot Analysis

For protein analysis, total lysates were extracted from cells or tissues using RIPA buffer supplemented with protease and phosphatase inhibitors. Protein concentration was quantified via a BCA assay. Equal protein quantities were resolved by SDS-PAGE and transferred to PVDF membranes (Millipore). The membranes were subsequently blocked with 5% non-fat milk in TBST and incubated overnight at 4°C with the indicated primary antibodies. Following incubation with HRP-conjugated secondary antibodies (see Supplementary Table 2 for details), immunoreactive bands were visualized using an ECL detection reagent and quantified by densitometry with ImageJ software.

Flow Cytometry and Fluorescence Probes

Mitochondrial superoxide was detected using MitoSOX Red (MCE). General cellular ROS was measured with a Dihydroethidium (DHE) probe (KeyGEN BioTECH). Mitochondrial membrane potential was assessed using a JC-1 kit (Beyotime). Labile iron (Fe^{2+}) was detected with FerroOrange (Dojindo), and lipid peroxidation was visualized with Liperfluo (Dojindo). Cells were stained according to the manufacturers' protocols and analyzed on a CytoFLEX flow cytometer (Beckman Coulter). MDA levels and the ratio of GSH/GSSG were measured using commercial kits from Beyotime, following the manufacturer's instructions.

RNA Isolation and Quantitative RT-PCR

Total RNA was isolated from cultured cells or tissue samples using TRIzol reagent (Invitrogen). Following the elimination of residual genomic DNA, complementary DNA (cDNA) was synthesized from 1 μg of total RNA utilizing the HiScript III RT SuperMix (Vazyme). The quantification of specific transcripts was subsequently performed by qPCR on a QuantStudio 5 system (Applied Biosystems) with SYBR Green Master Mix (Vazyme). Relative gene expression levels were determined using the $2^{-}\Delta\Delta Ct$ comparative threshold method, normalized to Actb (β-actin) as an endogenous reference gene. The specific primer sequences employed for amplification are provided in the Supplementary Materials (Table S5).

RNA Immunoprecipitation (RIP)

RNA immunoprecipitation (RIP) was conducted using the Magna RIP Kit (Millipore) in accordance with the manufacturer's guidelines. Cell lysates were subjected to immunoprecipitation with magnetic beads conjugated to either an anti-PGC-1α antibody (Proteintech, Cat# 66369-1-Ig) or a control IgG. Following stringent washing steps to remove non-specific binding, the co-precipitated RNA was isolated and subsequently quantified by RT-qPCR.

Chromatin Isolation by RNA Purification (ChIRP)

ChIRP was performed using the Magna ChIRP RNA Interactome Kit (Millipore). SH-SY5Y cells were cross-linked with 1% formaldehyde. Chromatin was sonicated to an average size of 200-500 bp. Biotinylated probes targeting LINC-EPS or LacZ (negative control) were hybridized with the chromatin lysate. The probe-chromatin complexes were captured with streptavidin magnetic beads. After washing and elution, the associated DNA was purified and analyzed by qPCR.

RNA Pull-down Assay

Biotinylated LINC-EPS and antisense control probes were transcribed *in vitro* (T7 RiboMAX, Promega). Probes were bound to streptavidin magnetic beads (Invitrogen) and incubated with SH-SY5Y cell lysates. After washing, bound proteins were eluted and analyzed by Western blotting.

Lentivirus Production and Infection

Lentiviral vectors for LINC-EPS overexpression (pLV-LINC-EPS) and knockdown (sh-LINC-EPS; target sequence: GCCCCCTGCCCTGCCCACTG) were constructed by Tsingke Biotechnology (Beijing, China). Empty vector (empty) and non-targeting shRNA (shNC) served as controls. Lentivirus was produced by co-transfecting 293T cells with the transfer plasmid and packaging plasmids (psPAX2, pMD2.G) using Lipofectamine 8000 (Thermo Fisher). Viral supernatants were collected, filtered (0.45 μm), and used to infect SH-SY5Y cells in the presence of polybrene (8 μg/mL). Stable cell lines were selected using puromycin (2 μg/mL; Sigma-Aldrich).

Dual-Luciferase Reporter Assay

The putative TBE was amplified from genomic DNA and inserted in either forward (TBE-F) or reverse (TBE-R) orientation downstream of a Ppargc1a promoter–luciferase cassette (2 kb upstream of the transcription start site, cloned into pGL3-basic; Promega). Control constructs included pGL3-

basic alone and pGL3 constructs containing TBE sequences without the Ppargc1a promoter. All plasmids were verified by Sanger sequencing.

SH-SY5Y cells were transiently co-transfected with a firefly luciferase reporter construct (500 ng) and a pRL-TK Renilla luciferase normalization control vector (50 ng; Promega), utilizing Lipofectamine 3000 (Invitrogen). Twenty-four hours post-transfection, cells were subjected to the indicated treatments prior to lysis. The luminescence from both luciferases was quantified using the Dual-Luciferase Reporter Assay System (Promega) with a GloMax 96 microplate luminometer. To control for transfection efficiency, firefly luciferase activity was normalized to the corresponding Renilla luciferase activity.

158 # Supplementary Table

159 **Table S1. The main reagents and kits**

Product Name	Supplier	Catalog Number (if available)
Chemicals and Inhibitors		
MPTP	Sigma-Aldrich (St. Louis, MO, USA)	M0896
MPP^+ iodide	Sigma-Aldrich (St. Louis, MO, USA)	D048
Ferrostatin-1	MCE (Monmouth Junction, NJ, USA)	HY-100579
Necrostatin-1	MCE (Monmouth Junction, NJ, USA)	HY-15760
Z-VAD-FMK	MCE (Monmouth Junction, NJ, USA)	HY-16658B
Mito-TEMPO	MCE (Monmouth Junction, NJ, USA)	HY-112879
Visomitin (SkQ1)	MCE (Monmouth Junction, NJ, USA)	HY-112130
Cell Culture and Transfection		
DMEM, high glucose	Servicebio (Wuhan, China)	G4510
Fetal Bovine Serum (FBS)	Gibco (Grand Island, NY, USA)	10099141
Penicillin-Streptomycin Solution	Beyotime (Shanghai, China)	C0222

Opti-MEM I Reduced Serum Medium	Gibco (Grand Island, NY, USA)	31985070
Molecular Biology Kits		
AccuRT-SuperMix	Accurate Biology (Hunan, China)	AG11728
RIPA Lysis Buffer	KeyGEN BioTECH (Jiangsu, China)	KGP702
BCA Protein Assay Kit	Solarbio (Beijing, China)	PC0020
SDS-PAGE Gel Preparation Kit	U-landy (Suzhou, China)	
Protein A/G Magnetic Beads	MCE (Monmouth Junction, NJ, USA)	HY-K0202
Western Blotting Reagents		
PVDF Membrane, 0.45 µm	Millipore (Billerica, MA, USA)	IPVH00010
NcmColor Prestained Protein Ladder	NCM Biotech (Suzhou, China)	
Non-fat Dry Milk	Lanjieke Tech (China)	
Tris, Glycine, SDS, Tween-20	Solarbio (Beijing, China)	
Fluorescent Probes and Assay Kits		
MitoSOX™ Red Indicator	MCE (Monmouth Junction, NJ, USA)	HY-D0942 (from Invitrogen)

DHE (ROS) Detection Kit	KeyGEN BioTECH (Jiangsu, China)	KGAF019
FerroOrange	Dojindo (Kumamoto, Japan)	F374
Liperfluo	Dojindo (Kumamoto, Japan)	L248
Mitochondrial Membrane Potential Kit (JC-1)	Beyotime (Shanghai, China)	C2006
MDA Assay Kit	Beyotime (Shanghai, China)	S0131S
GSH and GSSG Assay Kit	Beyotime (Shanghai, China)	S0053

Table S2 The main antibodies

Antibody name	Manufacturer	Product Number	Dilution ratio （WB）	Dilution ratio （IF/IP）	Dilution ratio (IP/RIP/MERIP)
TH	Servicebio	GB11181-100	1:500	1:200	
TH	Servicebio	GB12181-100	1:500	1:200	
xCT	Servicebio	GB115276-100	1：500		
α-Syn	CST	45083SF	1:1000	1:200	
p53	CST	9238	1:1000		
Bcl-2	CST	3948	1:1000		
β-Actin	CST	3700S	1:1000		

GPX4	HUABIO	AB_3070665	1:10000	1:100	
GPX4	ABclonal	A13309	1:1000	1:100	
CQ10B	UpingBio	YP-mAb-18654	1:1000		
CQ10A	UpingBio	YP-mAb-09554	1:1000		
FTL	Proteintech	10727-1-AP	1:1000		
FSP1	Proteintech	20886-1-AP	1:1000		
Bax	CST	2772	1:1000		
Gasdermin D	CST	39754	1:1000		
GFAP	Abcam	ab7260	1:1000		
Iba1	WAKO	019-19741	1:1000		
ASC	CST	67824	1:1000		
Phospho-RIP1 (Ser166)	CST	31122	1:1000		
Phospho-RIP3 (Ser227)	CST	91702	1:1000		
PGC-1α	Proteintech	66369-1-Ig	1:1000		1:25
ZBP1	Proteintech	13285-1-AP	1:1000		
RIPK1	CST	3493	1:1000		
RIPK3	CST	15828	1:1000		

NLRP12	Cusabio	CSB-PA015867GA01HU	1:1000
CL-Casp1	CST	89332	1:1000
Casp1	CST	83383	1:1000
CL-GSDMD	CST	10137	1:1000
CL-Casp3	CST	9664	1:1000
Casp3	CST	14220	1:1000
p-MLKL	CST	37333	1:1000
MLKL	CST	26539	1:1000
CL-Casp8	CST	8592	1:1000
Casp8	CST	4790	1:1000
NLRP3	CST	15101	1:1000
HRP-conjugated Goat Anti-Rabbit IgG(H+L)	Proteintech	SA00001-2	1:10000
HRP-conjugated Goat Anti-Mouse IgG(H+L)	Proteintech	SA00001-1	1:10000
Multi-rAb™	Proteintech	RGAR011	1:10000

Polymer HRP-Goat Anti-Rabbit			
Multi-rAb™ CoraLite® Plus 488-Goat Anti-Rabbit	Proteintech	RGAR002	1:200
Multi-rAb™ CoraLite® Plus 488-Goat Anti-Mouse	Proteintech	RGAM002	1:200
Multi-rAb™ CoraLite® Plus 594-Goat Anti-Rabbit	Proteintech	RGAR004	1:200
Multi-rAb™ CoraLite® Plus 594-Goat Anti-Mouse	Proteintech	RGAM004	1:200
Multi-rAb™ CoraLite® Plus 647-Goat Anti-Rabbit	Proteintech	RGAR005	1:200

Multi-rAb™ CoraLite® Plus 647-Goat Anti-Mouse	Proteintech	RGAM005	1:200

Table S3: Human blood sample information

HD

Sample	Gender: 1 male and 2 females	Age
1	2	39
2	2	87
3	2	53
4	1	38
5	1	38
6	1	72
7	1	59
8	2	50
9	2	75
10	2	70

11	2	56
12	1	64
13	2	62
14	1	48
15	1	76
16	2	71
17	1	42
18	2	87
19	1	66
20	2	63
21	2	56
22	2	57
23	1	57
24	2	42
25	1	57
26	2	56
27	1	56
28	1	25

29	1	56
30	1	78

PD

Sample	Course of the disease (years)	Gender: 1 male and 2 females	Age	UPDRS-III
1	5	1	63	33
2	11	1	77	18
3	4.5	2	68	19
4	0.5	2	76	13
5	2	1	66	19
6	7	2	65	12
7	1	2	76	21
8	11	1	72	20
9	4	1	62	20
10	1.5	1	70	32
11	2	2	71	16
12	5	2	63	28
13	9	1	70	23
14	1	1	60	2
15	6	1	60	6
16	3	1	65	6
17	4	2	67	29
18	1	1	67	16
19	2	2	63	15
20	1.5	2	52	26

21	1	1	78	8
22	10	2	77	28
23	3	1	60	12
24	1	1	61	8
25	2	1	53	13
26	2	2	62	13
27	1	1	75	10
28	6	2	74	12
29	10	1	80	26
30	11	1	82	29

Table S4-1 Primer sequences for mouse tail identification

Primer name	Upstream sequence	Downstream sequence
LINC-EPS-1	GCAGACAGGCGTGGACATTC ATTCT	GCTTGTACTCGCCTCTTCTCTGCA A
LINC-EPS-2	TCACTGAATACACAGGCTGCT GCAA	GCTTGTACTCGCCTCTTCTCTGCA A
Primer name	Upstream sequence	Downstream sequence
LINC-EPS$^{f/f}$	CCTTAACAAATGTGGTCGCAT ACC	CAACCCCAACCAGCAGATAAAG
DAT^{Cre}	TGGCTGTTGGTGTAAAGTGG	GGACAGGGACATGGTTGACT

Table S4-2 PCR Sample Loading System

Component	Volume
Forward Primer (10 μM)	1 μL
Reverse Primer (10 μM)	1 μL
DNA	1.5 μL
2×Taq PCR Master Mix	12.5 μL
ddH2O	9 μL

Table S4-3 PCR Program

Stage	temperature	time	cycle number
Stage 1	95℃	5 min	1
	95℃	30 s	
Stage 2	60℃	30 s	35
	72℃	45 s	
Stage 3	Default Settings of the instrument		1

Table S5 The primer sequences

Primer name	Upstream sequence	Downstream sequence
GAPDH	AAATCCCATCACCATCTTCCAG	AGGGGCCATCCACAGTCTTCT
m-LINC-EPS	GCGCACTTCTCTCATCTGTG	TCAGCTGTAGGATGGGAGGT
h-LINC-EPS	CGCATTAATGGGGGCATTCG	CTAAACCGTTTTCCCCGC
Bdnf	ATGGCGTTTCTCCGAAGCAT	TCCGCCCTATAAGCATCTTGA
Nt3	CCGTGGCATCCAAGGTAACAA	GCAGTTCGGTGTCCATTGC
Nt4	CTGTGTGCGATGCAGTCAGT	TGCAGCGGGTTTCAAAGAAGT
Gdnf	GGCAGTGCTTCCTAGAAGAGA	AAGACACAACCCCGGTTTTTG
Igf	GCTCTTCAGTTCGTGTGTGGA	GCCTCCTTAGATCACAGCTCC
Ngf	GGCAGACCCGCAACATTACT	CACCACCGACCTCGAAGTC
Fgf	CAGGCGGAGGCAGCTATAC	CCTGGTTCCCTGGATAGTACC
PGC-1α	GCTTTCTGGGTGGACTCAAGT	GAGGGCAATCCGTCTTCATCC
NRF1	AGGAACACGGAGTGACCCAA	TATGCTCGGTGTAAGTAGCCA
NRF2	TCAGCGACGGAAAGAGTATGA	CCACTGGTTTCTGACTGGATGT
TFAM	ATGGCGTTTCTCCGAAGCAT	TCCGCCCTATAAGCATCTTGA
MFN1	TGGCTAAGAAGGCGATTACTGC	TCTCCGAGATAGCACCTCACC
MFN2	CTCTCGATGCAACTCTATCGTC	TCCTGTACGTGTCTTCAAGGAA

OPA1	TGTGAGGTCTGCCAGTCTTTA	TGTCCTTAATTGGGGTCGTTG
DNM1L	CTGCCTCAAATCGTCGTAGTG	GAGGTCTCCGGGTGACAATTC
FIS1	AGCGGGATTACGTCTTCTACC	CATGCCCACGAGTCCATCTTT
MFF	ACTGAAGGCATTAGTCAGCGA	TCCTGCTACAACAATCCTCTCC
PINK1	CCCAAGCAACTAGCCCCTC	GGCAGCACATCAGGGTAGTC
Parkin	GTGTTTGTCAGGTTCAACTCCA	GAAAATCACACGCAACTGGTC
MT-ND1	GGCTATATACAACTACGCAAAGGC	GGTAGATGTGGCGGGTTTTAGG
MT-ND3	CCACAACTCAACGGCTACATAGAAA	GGGTAAAAGGAGGGCAATTTCTAGA
MT-ND6	CAAACAATGTTCAACCAGTAACCACTAC	ATATACTACAGCGATGGCTATTGAGGA
NDUFA3	GGGGCCTCGCTGTAATTCTG	GACGGGCACTGGGTAGTTG
MT-CYB	ATCACTCGAGACGTAAATTATGGCT	TGAACTAGGTCTGTCCCAATGTATG
ATP5F1A	GTATTGCCCGCGTACATGG	AGGACATACCCTTTAAGCCTGA
MT-ATP6	TAGCCATACACAACACTAAAGGACGA	GGGCATTTTTAATCTTAGAGCGAAA

Table S6 The English abbreviations and full names that appear in the article

Abbreviation	Full name
CHIRP	Chromatin Isolation by RNA Purification
Act-D	Actinomycin D
ChIP	Chromatin Immunoprecipitation
CHX	Cycloheximide
CCK-8	Cell Counting Kit-8
DAT	Dopamine Transporter
DAPI	4’,6-diamidino-2-phenylindole
DA neurons	Dopaminergic neurons
DFO	Deferoxamine
Fe^{2+}	Ferrous iron
FISH	Fluorescence *in situ* hybridization
GSEA	Gene Set Enrichment Analysis
GPX4	Glutathione peroxidase 4
GSH	Glutathione (reduced form)

GSSG	Glutathione disulfide (oxidized form)
IF	Immunofluorescence
IP	Immunoprecipitation
JC-1	5,5',6,6'-tetrachloro-1,1',3,3'-tetraethylbenzimidazolylcarbocyanine iodide
KEGG	Kyoto Encyclopedia of Genes and Genomes
KO	Knockout
LINC-EPS	Long intergenic non-coding RNA-Enhancer of Polycomb-like protein
MDA	Malondialdehyde
MMP	Mitochondrial membrane potential
MPP^+	1-methyl-4-phenylpyridinium
MPTP	1-methyl-4-phenyl-1,2,3,6-tetrahydropyridine
mitoROS	Mitochondrial reactive oxygen species
NRF1	Nuclear respiratory factor 1
NRF2	Nuclear factor erythroid 2-related factor 2

oeLINC-EPS	Overexpression of lncRNA-EPS
oeNC	Overexpression negative control
PD	Parkinson’s disease
PGC-1α	Peroxisome proliferator-activated receptor-gamma coactivator 1-alpha
qRT-PCR	Quantitative real-time polymerase chain reaction
RIP	RNA Immunoprecipitation
ROS	Reactive oxygen species
SEM	Standard error of the mean
shLINC-EPS	Short hairpin RNA targeting lncRNA-EPS
shNC	Non-targeting control short hairpin RNA
SNpc	Substantia nigra pars compacta
TBE	T-box element
TEAD	TEA domain family member
TEM	Transmission electron microscopy

TH	Tyrosine hydroxylase
TFAM	Mitochondrial transcription factor A
WT	Wild-type
xCT	Solute Carrier Family 7 Member 11 (SLC7A11)
FSP1	Ferroptosis suppressor protein 1 (AIFM2)
FTL	Ferritin Light Polypeptide
ZLN005	A specific agonist of PGC-1α
